# Adipose Tissue Dysfunction in Metabolic Dysfunction-Associated Steatotic Liver Disease

**DOI:** 10.3390/cells15151377

**Published:** 2026-07-30

**Authors:** Andrew John Legaspi Cruz, Liyun Yuan

**Affiliations:** 1 Department of Internal Medicine, Los Angeles County + USC Medical Center, Los Angeles, CA 90033, USA; andrewjohn.cruz@med.usc.edu; 2 Division of Gastrointestinal and Liver Diseases, Keck School of Medicine of USC, Los Angeles, CA 90033, USA

**Keywords:** MASLD, MASH, visceral adipose tissue, adipose tissue insulin resistance, personal fat threshold, sarcopenic visceral obesity, lean MASLD, myosteatosis, body composition, FGF21

## Abstract

**Highlights:**

**What are the main findings?**

- Adipose tissue dysfunction in MASLD is characterized by four complementary human-measurable dimensions (depot quantity, subcutaneous adipose tissue fibrogenesis, adipose tissue insulin resistance, and adipokine profile), and each maps onto identifiable body-composition phenotypes such as lean MASLD and sarcopenic visceral obesity.
- Recent MASH therapies sort into three mechanistic categories: adipose-mediated (pioglitazone, FGF21 analogs), weight-mediated (semaglutide, tirzepatide, lifestyle, bariatric surgery), and liver-directed (resmetirom). Each interacts differently with adipose biology.

**What are the implications of the main findings?**

- Integrating adipose function, fat distribution, and muscle quality alongside conventional liver-focused endpoints supports risk stratification and personalized therapy selection in MASLD.
- Adipose–liver–muscle framing motivates upstream-plus-downstream combination trials that pair mechanism classes across the axis rather than adding same-class agents.

**Abstract:**

Metabolic dysfunction-associated steatotic liver disease (MASLD) is clinically heterogeneous. Patients with similar body mass index, waist circumference, or noninvasive fibrosis stratum may differ markedly in histologic activity, fibrosis trajectory, and progression risk. Adipose tissue dysfunction helps explain this variation. Adipose dysfunction has been characterized by direct depot quantification, measurement of subcutaneous adipose tissue fibrogenesis, adipose tissue insulin resistance, and circulating adipokine profiles. These measures capture different features of adipose biology and have each been linked to liver injury or fibrosis severity. Clinically recognizable body-composition phenotypes—lean MASLD, sarcopenic visceral obesity, myosteatosis, and the hypertriglyceridemic waist—differ in accessibility, mechanistic specificity, and prognostic value. Recent therapeutic developments in metabolic dysfunction-associated steatohepatitis (MASH), including resmetirom, semaglutide, tirzepatide, fibroblast growth factor 21 (FGF21) analogs, and older adipose-directed agents such as pioglitazone, illustrate the importance of distinguishing adipose-mediated, weight-mediated, and liver-directed mechanisms. This review summarizes the literature on adipose tissue dysfunction in MASLD, the body-composition phenotypes associated with it, and recent therapeutic data in the context of adipose–liver–muscle biology.

## 1. Introduction

Metabolic dysfunction-associated steatotic liver disease (MASLD) is the most common chronic liver condition worldwide. Large meta-analyses estimate a global adult prevalence near one-third—30.05% (95% CI 27.88–32.32) across 92 studies in the analysis by Younossi and colleagues and 32.4% in the systematic review by Riazi and colleagues—with both reports describing rising prevalence over time [1,2]. The 2023 Delphi nomenclature anchors the diagnosis to cardiometabolic dysfunction [3]. AASLD practice guidance addresses clinical assessment and management in the current nomenclature transition [4]. An ADA consensus report emphasizes screening and early intervention in people with diabetes [5]. The spectrum extends from steatosis to metabolic dysfunction-associated steatohepatitis (MASH), progressive fibrosis, cirrhosis, and hepatocellular carcinoma. Progression is uneven: some lean patients have advanced disease, whereas some patients with severe obesity remain histologically mild for years [6,7]. Lean metabolic dysfunction-associated fatty liver disease (MAFLD) has been associated with higher liver-related mortality than overweight or obese MAFLD in a large pooled analysis, despite similar all-cause mortality and broadly similar conventional metabolic profiles [8].

Adipose tissue determines whether surplus lipid is buffered safely, mobilized appropriately, or redirected to ectopic sites. Subcutaneous adipose tissue (SAT) is the main protective lipid reservoir. When SAT expandability is exceeded or adipose function declines, lipid flux increases toward visceral and ectopic depots, including the liver, skeletal muscle, and pancreas [9,10,11,12,13]. The adiposity level at which this redirection occurs varies among individuals and has been described as the personal fat threshold [6,14]. Below that threshold, adipose tissue can buffer lipid without overt ectopic accumulation or clinically apparent metabolic dysfunction; above it, ectopic lipid deposition and MASLD-associated metabolic abnormalities become more likely. This framework is consistent with lean MASLD presenting as the same biology occurring at lower body weight rather than as a distinct disease entity.

Several recent reviews have addressed parts of the adipose–liver–muscle axis. Marjot and colleagues reviewed skeletal muscle in MASLD pathogenesis [15], and Henin and colleagues provided a systematic review of myosteatosis [16]. Other reviews have cataloged organokine signaling between liver and peripheral organs [17]. Others have addressed systemic MASLD complications involving the heart, muscle, and kidney [18], or the cellular consequences of hepatic fat accumulation [19]. Data-driven cluster analyses also support biological heterogeneity within MASLD. Raverdy and colleagues applied unsupervised clustering to six routine clinical variables (age, body mass index (BMI), triglycerides, HbA1c, ALT, and LDL cholesterol) in a French bariatric-surgery cohort, with replication across independent biopsy cohorts. They reported two MASLD subtypes: a liver-specific cluster with high ALT and patatin-like phospholipase domain-containing 3 (PNPLA3) risk-allele enrichment, and a cardiometabolic cluster with high HbA1c and triglycerides that carried higher cardiovascular and incident-diabetes risk [20].

A brief note on terminology: throughout this review, we use MASLD to describe hepatic steatosis with at least one cardiometabolic risk factor as defined by the 2023 Delphi consensus [3], and MASH to describe the necro-inflammatory subtype with hepatocyte ballooning and lobular inflammation. We use the legacy terms NAFLD, NASH, and MAFLD when discussing studies published under those definitions. The 2023 nomenclature anchors diagnosis to cardiometabolic dysfunction, and this mandatory metabolic framing modestly reshapes interpretation of earlier anatomically defined fatty-liver cohorts: analyses in East Asian and Western populations have shown near-complete overlap between NAFLD and MASLD [1,2], so most legacy data can be interpreted forward, but a small subset of steatosis without cardiometabolic risk falls outside MASLD and is now classified separately. Where the distinction matters for a specific study or biomarker interpretation, we flag it explicitly.

**Figure 1:**
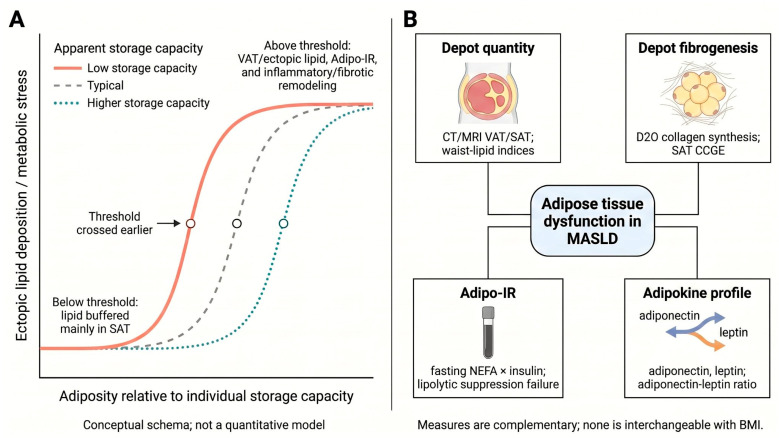
Adipose tissue dysfunction in MASLD. (**A**) The personal fat threshold as a conceptual schematic rather than a quantitative model: patients differ in apparent storage capacity, and MASLD can occur at lower body weight when the threshold is crossed early. (**B**) Complementary approaches to measuring adipose dysfunction: depot quantity by imaging or indirect waist–lipid indices, depot fibrogenesis by in vivo collagen synthesis or SAT composite collagen gene expression, adipose tissue insulin resistance (Adipo-IR = fasting non-esterified fatty acids (NEFA) × fasting insulin), and adipokine profile. These domains overlap but are not interchangeable with BMI [6,11,21,22,29,30]. Created in BioRender. Cruz, A. J. (2026) https://BioRender.com/m9b4cj5 (accessed on 25 July 2026).

This review synthesizes human MASLD studies that have characterized adipose dysfunction through depot quantity, adipose fibrogenesis, adipose tissue insulin resistance, and adipokine profile, and integrates these measurements with composite body-composition phenotypes and recent therapeutic trial data organized by adipose-mediated, weight-mediated, and liver-directed mechanisms (Figure 1). Section 2 addresses the personal fat threshold and adipose tissue capacity, including recent evidence on adipose fibrogenesis [21,22] and genetic variants that modify hepatic susceptibility. Section 3 reviews adipose dysfunction as measured in MASLD cohorts. Section 4 addresses body-composition phenotypes linked to liver and cardiometabolic outcomes, including lean MASLD, sarcopenic visceral obesity, myosteatosis, and the hypertriglyceridemic waist. Section 5 considers recent therapeutic developments in MASH organized by adipose-mediated, weight-mediated, and liver-directed mechanisms.

### Literature Identification

We identified sources through targeted searches of PubMed and Google Scholar (from database inception to November 2025), using combinations of the terms “MASLD,” “MASH,” “NAFLD,” “NASH,” “MAFLD,” “adipose tissue,” “visceral adipose tissue,” “subcutaneous adipose tissue,” “adipose insulin resistance,” “adipokine,” “personal fat threshold,” “lean MASLD,” “sarcopenic obesity,” “myosteatosis,” “body composition,” “resmetirom,” “semaglutide,” “tirzepatide,” “pioglitazone,” “FGF21,” “bariatric surgery,” and “neutrophil extracellular trap.” Priority was given to (i) human cohort studies and randomized trials that reported adipose or body-composition measurements alongside liver histology or noninvasive fibrosis measures, (ii) mechanistic studies that established biological plausibility for identified associations, and (iii) recent guidelines and consensus statements. Reference lists of retrieved articles were hand-searched for additional relevant sources. Legacy NAFLD/NASH/MAFLD studies were retained where their findings remain relevant under current MASLD/MASH definitions.

## 2. The Adipose Tissue Capacity

Adipose tissue capacity has been proposed to address the clinical phenotype of adipose dysfunction: each person has a maximal adiposity storage level above which lipid is increasingly redirected to visceral adipose tissue (VAT) and ectopic sites, where it contributes to lipotoxicity and insulin resistance. Capacity varies substantially among individuals because genetics, fat distribution, adipocyte biology, and adipose remodeling differ even among people with similar BMI [6,14]. In the ReTUNE study [23], 20 patients with type 2 diabetes and BMI < 27 kg/m^2^ underwent repeated 5% weight-loss cycles with metabolic phenotyping at each stable-weight plateau through 12 months. After modest weight loss, BMI fell from 24.8 to 22.5 kg/m^2^, liver and pancreatic fat decreased, and most participants achieved diabetes remission with pathophysiologic changes similar to those seen in obese remission from earlier studies in the same program [23]. These findings are consistent with type 2 diabetes occurring at conventionally normal BMI when an individual’s storage capacity is exceeded. Lean MASLD has been interpreted similarly, as the same metabolic process emerges at lower body weight when the storage threshold is exceeded [6,8,9]. Saponaro and colleagues studied 32 non-diabetic, biopsy-proven non-alcoholic fatty liver disease (NAFLD) patients (20 with non-alcoholic steatohepatitis [NASH], 12 with non-alcoholic fatty liver [NAFL]) and eight lean controls using MRI quantification of liver, visceral, and abdominal subcutaneous fat, palmitate flux measurement, and biopsy histology [11]. NASH patients had higher visceral fat than NAFL patients despite similar BMI. Palmitate flux to the liver also predicted intrahepatic triglyceride accumulation and the histologic pattern of NASH. Elevated VAT, increased free fatty acid (FFA) flux, and steatohepatitis at preserved BMI are consistent with inadequate SAT buffering for a given individual.

Adipose tissue capacity has long been framed in terms of expandability: lipotoxicity emerges when SAT can no longer accommodate additional lipid safely [24]. The limit may be functional rather than absolute, with evidence that adipose tissue undergoes collagen remodeling in a way that limits further healthy expansion. SAT collagen remodeling has been demonstrated in obese NAFLD patients before lipogenic capacity is exhausted [21]. This fibrogenesis evidence is described in Section 3.2 [21,22].

Several questions remain. Direct measurement of SAT collagen synthesis has been performed in relatively small human cohorts. The degree of fibrogenesis at which liver disease emerges is unknown. The relationship between SAT fibrogenesis and other measures of adipose dysfunction—particularly adipose tissue insulin resistance (Adipo-IR)—has not been examined directly in paired-measurement studies. These limitations argue for caution: SAT fibrogenesis is best regarded as an emerging research measure of adipose dysfunction rather than a clinical biomarker at present.

The PNPLA3 I148M variant (rs738409), a missense substitution in the encoded protein, is associated with increased risk across the MASLD spectrum, from steatosis to cirrhosis and hepatocellular carcinoma [25,26]. The variant reduces triglyceride hydrolase activity [27] and accumulates on hepatic lipid droplets, where it impairs lipid mobilization [28]. The transmembrane 6 superfamily member 2 (TM6SF2) E167K variant (rs58542926) impairs very-low-density lipoprotein (VLDL) assembly and export, increasing hepatic triglyceride retention while lowering circulating low-density lipoprotein (LDL) cholesterol [25]. MBOAT7 rs641738 affects phospholipid remodeling and has been associated with steatohepatitis and fibrosis in MASLD and with alcohol-associated liver disease [25]. Hydroxysteroid 17-beta dehydrogenase 13 (HSD17B13) truncating variants are associated with reduced risk of progressive liver disease, including among PNPLA3 risk-allele carriers, and encode a hepatocyte lipid-droplet protein with retinol dehydrogenase activity [25]. Glucokinase regulator (GCKR) P446L (rs1260326) increases glucokinase activity, hepatic de novo lipogenesis, and serum triglycerides and has been associated with steatosis [25]. These variants modify hepatic susceptibility and may lower the adiposity burden needed for steatosis, inflammation, or fibrosis to appear.

## 3. Adipose Tissue Dysfunction in MASLD

Human MASLD studies have measured adipose tissue dysfunction through several partly overlapping approaches: depot quantity, adipose fibrogenesis, adipose tissue insulin resistance, and adipokine profile. Each captures a different aspect of adipose biology. The choice of method often determines which abnormality is detected and how strongly it appears to relate to liver outcomes.

### 3.1. Visceral Adipose Tissue Volume

VAT volume is the longest-studied adipose measurement in MASLD. VAT can be quantified directly from CT or MRI cross-sections, and many cohorts have examined its relation to histologic and imaging-defined liver disease. In a biopsy-proven NAFLD cohort of 324 patients and 132 controls, Yu and colleagues showed that VAT area at the L3 vertebral level correlated with histologic NASH severity and fibrosis stage independent of BMI [31]. In the Saponaro cohort, MRI-measured visceral fat was the body-composition variable most strongly associated with palmitate flux to the liver and with NASH histology [11]. Across available studies, direct VAT measurement by CT or MRI has shown a more consistent association with MASLD severity than total body fat or BMI alone.

VAT volume, however, does not fully distinguish quantity from biology. Two patients with the same VAT area may differ in depot lipolysis, inflammatory state, and endocrine output. Indirect composite indices attempt to capture this functional aspect. The visceral adiposity index (VAI), which combines anthropometric and lipid variables, has been associated with NAFLD and fibrosis in a systematic review and meta-analysis [32]. These indices are accessible and useful for population-level risk context, but they conflate depot size with adipose function and are less specific than direct imaging or mechanistic adipose measures.

VAT and SAT differ developmentally and functionally, with consequences for MASLD biology. Experimental lineage-tracing studies by Chau and colleagues suggest that many visceral adipocytes derive from Wt1-expressing mesothelial precursors, whereas subcutaneous adipocytes do not [33]. Depot-specific progenitor programs across visceral and subcutaneous fat have been reviewed by Tchkonia and colleagues [34]. VAT adipocytes generally show higher basal and catecholamine-stimulated lipolytic activity and weaker insulin-mediated lipolytic suppression than SAT adipocytes [35]. Portal venous drainage from mesenteric VAT contributes to hepatic FFA exposure, although systemic FFA flux also remains important; this is the portal-delivery component of the portal hypothesis [36]. Donnelly and colleagues used stable-isotope methods in NAFLD and estimated that approximately 59% of intrahepatic triglyceride derived from circulating non-esterified fatty acids, predominantly of adipose origin, with the remainder from de novo lipogenesis (~26%) and diet (~15%) [37]. Within both VAT and SAT, expansion can occur by adipocyte hyperplasia or hypertrophy; hypertrophic expansion is linked to inflammation, fibrosis, and metabolic dysfunction independent of total adipose mass [34]. These differences help explain why VAT quantity relates more closely to liver outcomes than BMI, while still leaving important biology unmeasured.

### 3.2. Adipose Tissue Fibrogenesis

Beals and colleagues tested cellular correlates of adipose tissue capacity in vivo by measuring SAT collagen synthesis and lipogenesis with deuterated-water labeling in 12 lean controls, 24 obese controls without NAFLD, and 25 obese individuals with NAFLD [21]. Obese NAFLD participants had higher SAT collagen fractional synthesis rates than obese controls without NAFLD (*p* < 0.05), whereas lipogenic capacity did not differ between groups. The finding suggested that obese individuals with NAFLD had not simply exhausted lipogenic capacity; rather, their SAT was depositing extracellular matrix at higher rates, in keeping with fibrotic remodeling under chronic caloric overload. Bilson and colleagues later reported convergent transcriptional evidence: a composite collagen gene-expression signature derived from SAT biopsies was elevated in obese NAFLD patients and associated with clinically significant liver fibrosis [22]. These studies support SAT fibrogenesis as a candidate human-measurable marker of adipose dysfunction.

Adipose fibrogenesis is not yet clinically usable. Direct quantification requires deuterated-water labeling, which is technically demanding and not widely available, or tissue biopsy, which is invasive. The SAT composite collagen gene-expression signature described by Bilson and colleagues is promising but has not been deployed at scale across MASLD cohorts [22]. No noninvasive imaging method has been validated specifically for SAT fibrogenesis. The measurement remains a research tool that can complement more accessible readouts such as VAT, Adipo-IR, and adipokines. Emerging biomarker directions may partially overcome these bottlenecks in the future. Serum collagen-turnover neoepitopes, such as PRO-C3 (a marker of type III collagen formation) and C3M (a marker of collagen degradation), have shown early promise as circulating markers of tissue fibrogenesis in liver and adipose research; specialized magnetic resonance elastography and diffusion-weighted sequences are also being adapted for adipose tissue characterization. These approaches have not yet been validated for adipose-specific fibrogenesis in MASLD cohorts, but they represent testable avenues for noninvasive quantification.

Adipose fibrosis develops as expanding adipocytes interact with infiltrating immune cells and accumulating extracellular matrix [38]. In chronic caloric excess, adipose macrophages shift toward pro-inflammatory states and form crown-like structures around dying adipocytes [39], producing cytokines and chemokines such as TNF-α, IL-6, and MCP-1 [38]. Hypoxia develops when adipocyte hypertrophy outpaces vascularization. Using a constitutively active hypoxia-inducible factor 1 alpha (HIF-1α) deletion mutant (HIF1α-ΔODD) targeted to adipocytes, Halberg and colleagues showed that HIF-1α drives a pro-fibrotic transcriptional program rather than the canonical angiogenic vascular endothelial growth factor (VEGF) response, with upregulation of lysyl oxidase (LOX), elastin, collagens I, III, and VI, tissue inhibitor of metalloproteinases 1 (TIMP1), and connective tissue growth factor (CTGF). Pharmacologic LOX inhibition with β-aminopropionitrile rescued the metabolic phenotype, identifying collagen cross-linking as a downstream effector [40]. Khan and colleagues showed in obese mice that deletion of collagen VI permits adipocyte expansion with paradoxically improved metabolic profile, supporting collagen VI deposition as a functionally important extracellular-matrix change that mechanically constrains adipocyte expansion rather than a passive scar [41]. TGF-β1 signaling through SMAD2/3 drives collagen production by adipose-tissue resident myofibroblast-like cells [38]. These experimental and translational observations provide the mechanistic background for the fibrogenesis signal measured in human SAT by Beals and Bilson [21,22].

### 3.3. Adipose Tissue Insulin Resistance (Adipo-IR)

Among functional adipose tissue metrics, Adipo-IR has the strongest evidence base. Adipo-IR is calculated as fasting plasma NEFA multiplied by fasting insulin [29]. In healthy adipose tissue, insulin suppresses lipolysis and reduces FFA release; in insulin-resistant adipose tissue, lipolytic suppression is incomplete, so NEFA remains elevated despite hyperinsulinemia. The product captures this failure as a single fasting measure. NEFA and insulin are obtainable in research and selected clinical settings, although NEFA is not part of routine laboratory testing in many health systems.

A landmark cohort study by Lomonaco and colleagues demonstrated a close association between Adipo-IR and the severity of hepatic steatosis, inflammation, and fibrosis. In 207 patients with NAFLD characterized by liver MR spectroscopy and euglycemic insulin clamp, with biopsy histology available in 136, Adipo-IR was higher in NAFLD than in obese controls without NAFLD and associated with histologic inflammation, ballooning, and fibrosis stage across Adipo-IR quartiles [29]. A later obese type 2 diabetes cohort from the same group reported higher Adipo-IR in NASH than in isolated steatosis (9.8 ± 1.0 vs. 5.9 ± 0.8, *p* < 0.03) [42]. Adipo-IR correlated with intrahepatic triglyceride content and hepatic insulin sensitivity and was higher in patients with type 2 diabetes and NAFLD than in those with NAFLD without diabetes. Two subsequent observations further highlight the utility of Adipo-IR in explaining disease heterogeneity. Bril and colleagues found that Adipo-IR was elevated in MASLD regardless of PNPLA3 genotype, but PNPLA3 risk-allele carriers developed steatosis and steatohepatitis at lower Adipo-IR values than non-carriers [43]. This pattern is consistent with adipose dysfunction and PNPLA3-mediated hepatic susceptibility acting through distinct mechanisms, and with the threshold framework introduced in Section 2. Second, a PIVENS follow-up study showed that pioglitazone produced an early reduction in Adipo-IR at 16 weeks that was not sustained at 96 weeks, whereas vitamin E improved liver histology without changing Adipo-IR. Although the early acute fall was not maintained, residual differences in Adipo-IR at 96 weeks still tracked histology, with change in Adipo-IR at 96 weeks associated with histologic improvement including fibrosis (*p* = 0.004) [44]. Kalavalapalli and colleagues [45] also reported in patients with type 2 diabetes and NAFLD that Adipo-IR independently predicted clinically significant fibrosis by vibration-controlled transient elastography (VCTE), whereas the homeostatic model assessment of insulin resistance (HOMA-IR) and cytokeratin 18 (CK-18) lost significance after adjustment.

**Figure 2:**
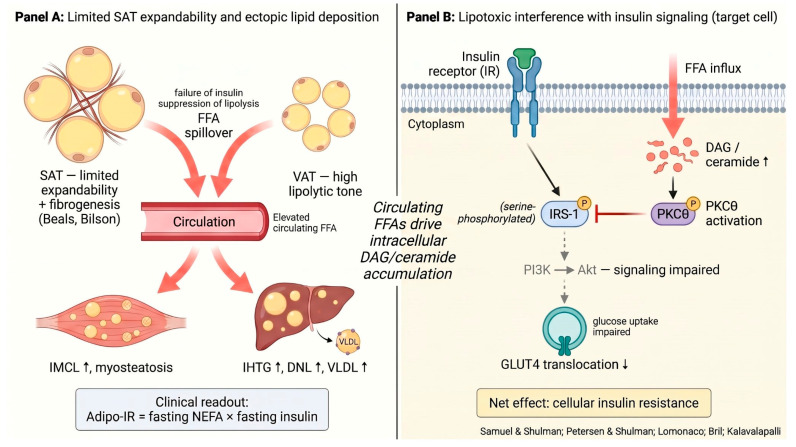
Adipose tissue insulin resistance and lipotoxic interference with insulin signaling. Arrows indicate the direction of lipid flux and signaling steps; (**A**) shows the whole-body state and (**B**) the intracellular mechanism. (**A**) When subcutaneous adipose tissue (SAT) expandability is limited and visceral adipose tissue (VAT) lipolytic tone is elevated, free fatty acids (FFAs) spill into the circulation as insulin fails to suppress lipolysis; ectopic lipid accumulates in liver (increased intrahepatic triglyceride [IHTG], de novo lipogenesis [DNL], very-low-density lipoprotein [VLDL] secretion) and skeletal muscle (intramyocellular lipid [IMCL], myosteatosis). The clinical readout of this in vivo state is Adipo-IR, calculated as fasting NEFA × fasting insulin. (**B**) Within target cells, FFA influx drives accumulation of lipid intermediates such as diacylglycerol (DAG) and ceramide, activating protein kinase C theta (PKCθ) and other novel-class PKC isoforms, which serine-phosphorylate IRS-1 and impair downstream PI3K/Akt signaling and GLUT4 translocation. The net effect is cellular insulin resistance [21,22,29,43,45,46,47]. Created in BioRender. Cruz, A. J. (2026) https://BioRender.com/4cau5l8 (accessed on 25 July 2026).

The molecular basis for adipose insulin resistance is well described [46,47]. In healthy adipocytes, insulin receptor activation signals through insulin receptor substrate 1 (IRS-1), phosphoinositide 3-kinase (PI3K), phosphatidylinositol (3,4,5)-trisphosphate (PIP3), and Akt. Akt promotes glucose transporter 4 (GLUT4) translocation and suppresses hormone-sensitive lipase and adipose triglyceride lipase, reducing FFA release during postprandial insulin exposure [46]. In obesity-associated adipose dysfunction, intracellular lipid intermediates such as diacylglycerol and ceramide activate stress and kinase pathways that impair IRS-1 and Akt signaling [47]. GLUT4 translocation falls, lipolytic suppression is incomplete, and fasting NEFA remains elevated at a given insulin concentration, the biochemical state that Adipo-IR captures. Adipose inflammation and fibrogenesis may coexist with these signaling abnormalities, but the DAG/ceramide pathway is best understood as an insulin-signaling mechanism rather than a complete explanation for macrophage recruitment or extracellular-matrix remodeling (Figure 2).

### 3.4. Adipokine Profile

The adipokine secretome reflects another aspect of adipose biology. Adipose tissue secretes signaling proteins whose circulating levels reflect depot health, systemic inflammation, and endocrine context. Adiponectin is adipocyte-derived and acts as an insulin-sensitizing, anti-inflammatory, and anti-fibrotic signal. Plasma adiponectin is inversely associated with hepatic steatosis, insulin resistance, and type 2 diabetes. In MASLD, circulating adiponectin levels are reduced, with a disproportionate decline in the high-molecular-weight (HMW) isoform, which mediates many of adiponectin’s key metabolic effects [10,30]. Leptin shows a different pattern: it is produced in proportion to adipose mass, is elevated in obesity and MASLD, and has been linked to hepatic stellate-cell activation and fibrogenesis [10,30]. The adiponectin-to-leptin ratio has been proposed as a composite marker, although its incremental value over individual adipokine measurements remains modest. Despite the growing number of candidate adipokines, adiponectin and leptin continue to demonstrate the most consistent associations with MASLD across diverse cohorts. Yet, both are influenced by factors beyond adipose dysfunction, including renal function, inflammatory state, sex hormone status, and total adiposity. Other adipose-derived or adipose-linked signals, including resistin, retinol-binding protein 4, and fetuin-A, have been studied in MASLD, but inter-assay variability and the absence of validated histology-linked cutpoints limit their clinical use [10,30].

Adiponectin signals through two receptors with distinct tissue distributions: AdipoR1 is expressed predominantly in skeletal muscle and AdipoR2 predominantly in liver [48]. Both signal through the adaptor protein APPL1 and activate AMPK. In hepatocytes, AMPK phosphorylates and inactivates acetyl-CoA carboxylase, lowering malonyl-CoA, disinhibiting carnitine palmitoyltransferase 1, and increasing mitochondrial fatty acid β-oxidation [49,50]. AMPK also suppresses SREBP1c expression, reducing hepatic de novo lipogenesis [50]. Reduced circulating adiponectin levels were found in MASLD and are linked to greater histologic and clinical disease severity, supporting a potential role for impaired adiponectin signaling in disease progression [10,30]. These measures capture overlapping but sometimes distinct dimensions of adipose tissue dysfunction and therefore do not always identify the same individuals. For example, increased VAT may occur in the absence of marked Adipo-IR, whereas elevated Adipo-IR can be observed despite relatively modest VAT accumulation. Similarly, hypoadiponectinemia and SAT fibrosis may provide additional, partially independent information. This incomplete concordance likely contributes to the heterogeneity of findings across body-composition studies that rely on different adipose tissue metrics.

### 3.5. Neutrophils and Neutrophil Extracellular Traps as a Converging Inflammatory-Fibrotic Axis

Adipose dysfunction sets the stage for hepatic injury, but fibrosis is not exclusively downstream of adipose signals. Several hepatic cell populations shape the fibrogenic response. Hepatic stellate cells are mesenchymal sinusoidal pericytes derived from septum transversum mesothelium [51] and are the principal source of fibrillar collagen in the injured liver. Innate immune cells, including Kupffer cells, monocyte-derived macrophages, and neutrophils, supply the inflammatory signals that activate them. Liver sinusoidal endothelial cells and platelets contribute additional non-immune signals. Neutrophils have emerged as important effectors in MASLD and MASH pathogenesis, contributing to hepatocyte injury and fibrogenesis through reactive oxygen species, cytokines, granule proteins (myeloperoxidase, neutrophil elastase, matrix metalloproteinases), and neutrophil extracellular traps (NETs), which are extruded webs of chromatin, histones, and granule enzymes released in response to microbial and sterile triggers [52].

Recent work has directly implicated NETs in MASH-associated fibrosis. Xia and colleagues reported that NETs promote MASH fibrosis in experimental models by reprogramming hepatic stellate cells through a Toll-like receptor 3 (TLR3)/cyclooxygenase-2 (COX-2)/prostaglandin E2 (PGE2) axis, and that DNase I treatment or peptidylarginine deiminase 4 (PAD4) deficiency attenuated MASH-fibrosis [53]. Babuta and colleagues showed in alcohol-accelerated MASH that NETs activate hepatic stellate cells and monocytes through NLRP3 sensing, with NET disruption reducing liver fibrosis in vivo [54]. Broader reviews of neutrophil biology in MASLD/MASH support neutrophils as active contributors to inflammatory and fibrotic signaling rather than passive markers of injury [52].

We use the term “NET formation” when speaking broadly; NETosis refers specifically to NET-associated cell death, whereas NET release can also occur through mechanisms that do not require cellular death. This distinction matters for mechanistic interpretation. From the adipose–liver perspective, adipose-derived free fatty acids, adipokine changes, and gut-derived signals may prime neutrophil activation; the resulting NET signaling then interacts with stellate-cell and macrophage programs to drive fibrosis. Neutrophil-directed pathways are not yet routinely measurable in clinical MASLD cohorts, but the experimental data motivate their inclusion in mechanistic frameworks.

## 4. Body-Composition Phenotypes in MASLD

MASLD cohorts have been phenotyped using imaging, anthropometric, laboratory, and physical-assessment methods. Four composite phenotypes recur as clinically informative markers of adipose-related risk: lean MASLD, sarcopenic visceral obesity, myosteatosis, and the hypertriglyceridemic waist. Their value depends on how adipose depots, muscle quality, and liver outcomes are measured.

### 4.1. Body-Composition Methods and What They Capture

CT and MRI directly segment visceral and subcutaneous depots and quantify muscle area and quality. Single-slice cross-sections at L3 or L4-L5 correlate well with whole-body measures (r approximately 0.90–0.94) [55,56], enabling opportunistic use of routine clinical scans, and AI-enabled pipelines now allow automated extraction at scale [57]. DXA (dual-energy X-ray absorptiometry) provides reliable appendicular lean mass and total fat estimates, but it does not segment VAT with the anatomic precision of CT or MRI and is limited for muscle quality. BIA (bioelectrical impedance analysis) is fast and inexpensive, but hydration status and device-specific equations affect its estimates. Surrogate waist–lipid indices, including VAI and related measures, are accessible without imaging but cannot separate depot quantity from depot function [9,32]. Liver endpoints add another source of heterogeneity: ultrasound, controlled attenuation parameter, transient elastography, magnetic resonance imaging proton density fat fraction (MRI-PDFF), magnetic resonance elastography, and biopsy do not measure the same biological feature. Apparent disagreement across studies often reflects endpoint mismatch rather than true biological contradiction. Table 1 summarizes the methods and their interpretive limits.

### 4.2. Visceral Adiposity in MASLD

Direct VAT measurement has shown one of the most consistent associations with MASLD severity [11,31]. Wakabayashi and colleagues reported in a Japanese longitudinal study that lean MASLD was heterogeneous over time, with risk profiles that did not track with BMI [7]. Zhang and colleagues reported that patients with moderate-to-severe steatosis had more visceral fat and more fibrosis than those with mild steatosis in a Chinese cohort [61]. A meta-analysis of 24 studies found that VAI predicted NAFLD with an AUC of 0.77 and NASH with an AUC of 0.73 [32]. The VAT signal is reproducible across populations and methods, although direct imaging remains more biologically specific than surrogate indices. A recurring observation across cohorts that combine VAT imaging with functional measures of adipose tissue biology (such as Adipo-IR, adiponectin levels, or markers of adipose fibrogenesis) is that visceral adiposity and adipose tissue dysfunction are correlated but do not completely overlap. When discordance is present, functional measures often demonstrate stronger associations with hepatic steatosis, steatohepatitis, fibrosis, or metabolic abnormalities than VAT quantity alone [11,22,29].

### 4.3. Skeletal Muscle Phenotypes in MASLD

The MASLD literature distinguishes two muscle properties that behave differently across cohorts: mass and quality. Mass-based sarcopenia, as defined by consensus criteria through low muscle strength, low muscle mass or quality, and reduced physical performance [62], has shown heterogeneous associations with MASLD outcomes [63]. In a Japanese NAFLD cohort, the lowest quartile of skeletal muscle-to-visceral-fat ratio was associated with higher steatosis and fibrosis severity, with liver stiffness rising 134% over 2–5 years in the lowest-ratio group [64]; comparable associations have been reported across additional Asian and U.S. cohorts using similar ratio metrics [65,66,67,68,69,70,71,72,73]. Sex modifies the effect: in biopsy-proven NAFLD, a lower appendicular skeletal muscle mass-to-visceral fat area ratio was inversely associated with NASH in men but not in women [74]. Pooled analyses confirm the heterogeneity. In a meta-analysis of 25 studies, sarcopenia was associated with higher odds of MASLD (OR 1.25, 95% CI 1.08–1.44) and fibrosis among MASLD patients (OR 1.49, 95% CI 1.03–2.14), but skeletal muscle index alone was not significantly associated (OR 1.02, 95% CI 0.91–1.15) [75]. A separate meta-analysis of 29 studies reported sarcopenia prevalence of 23.5% in MASLD and an independent association with all-cause mortality (aHR 1.59, 95% CI 1.33–1.91) [76]. The variability largely reflects how muscle is defined.

Emerging evidence suggests that myosteatosis (fat accumulation within skeletal muscle) is more consistently linked to hepatic outcomes than reduced muscle mass alone. Hsieh and colleagues reported that severe myosteatosis, but not mass-based sarcopenia, was associated with early MASH in biopsy-proven MASLD and with fibrosis progression in a longitudinal subset [58]. In lean MASLD, Kim and colleagues reported that visceral fat obesity combined with sarcopenia and/or myosteatosis identified a higher-risk phenotype [59]. In a large cross-sectional comparison of 18,154 participants using CT-defined normal-attenuation muscle area (NAMA)/total abdominal muscle area (TAMA) at L3, Kim and colleagues found that under the MASLD-only definition, both sarcopenia (adjusted OR 2.62, 95% CI 1.94–3.54) and myosteatosis (adjusted OR 1.75, 95% CI 1.52–2.02) were associated with disease [77]. A prospective interventional study reported that decreases in myosteatosis tracked improvement in liver stiffness [78]. MRI studies in trained and untrained individuals support the broader biological point: muscle size and intramuscular fat are separable phenotypes that respond differently to physical activity [79]. Preclinical NAFLD models also identify myosteatosis, rather than sarcopenia, as the muscle phenotype most closely associated with steatohepatitis severity [80]. Experimental work also supports bidirectional liver–muscle crosstalk, including hepatic steatosis-driven muscle atrophy and skeletal-muscle IRF4-FSTL1 signaling in NASH models [81,82].

Myosteatosis includes both intermyocellular adipose tissue (IMAT), visible between muscle fibers, and intramyocellular lipid (IMCL) within myofibers. IMCL is the more mechanistically consequential pool. Samuel, Shulman, and colleagues have described how IMCL-derived diacylglycerol activates PKCθ, which impairs IRS-1 signaling and reduces insulin-stimulated GLUT4 translocation [46,83]. Ceramide accumulation contributes to the same insulin-signaling defect through Akt inhibition. Petersen and colleagues showed using ^1^H-magnetic resonance spectroscopy that insulin-resistant lean offspring of patients with type 2 diabetes had elevated IMCL and impaired muscle glucose-6-phosphate accumulation during insulin infusion; subsequent stable-isotope studies showed redirection of postprandial energy toward hepatic de novo lipogenesis in lean insulin-resistant individuals [84,85]. Goodpaster and colleagues reported that low-density muscle on CT, an imaging correlate of myosteatosis, carries metabolic implications independent of muscle mass [86]. This distinction parallels the imaging literature: muscle quality has been more informative than muscle quantity alone in many MASLD cohorts.

### 4.4. Sarcopenic Visceral Obesity, Lean MASLD, and the Hypertriglyceridemic Waist

Sarcopenic visceral obesity (SVO) combines low muscle mass or quality with elevated visceral adiposity. In a MASLD cohort of 334 adults characterized by MRI body composition and magnetic resonance elastography, Elsabaawy and colleagues reported that SVO was present in 42.5% and independently predicted advanced fibrosis (adjusted OR 2.5, 95% CI 1.4–4.3, *p* = 0.002), with F3-F4 fibrosis in 58% of SVO versus 12% of non-SVO participants [60]. Chien and colleagues reported in 223 MASLD patients that DXA-defined sarcopenic obesity carried higher 10-year atherosclerotic cardiovascular disease (ASCVD) risk than MASLD without sarcopenic obesity (10.33% vs. 7.12%, *p* = 0.02), along with higher BMI, waist circumference, type 2 diabetes prevalence, hypertension, dyslipidemia, fibrosis-4 (FIB-4), and fatty liver index (FLI) scores [87]. In lean adults with type 2 diabetes, the combined phenotype of visceral obesity with sarcopenia carried the highest odds of lean NAFLD [69]. Recent reviews and a 2025 commentary have proposed recognizing SVO as a distinct clinical entity in MASLD, but diagnostic criteria are not yet standardized [88]. Representative studies linking body-composition phenotypes to MASLD outcomes are summarized in Table 2.

Lean MASLD describes patients who meet MASLD criteria at normal BMI [89]. Yet, lean MASLD patients often have elevated VAT relative to BMI-matched controls without MASLD [7,11], multi-tissue insulin resistance with elevated Adipo-IR [11], and reduced adiponectin [11,90]. In a meta-analysis pooling 10,013,382 individuals, lean MASLD was associated with higher liver-related mortality than overweight or obese MASLD despite broadly similar conventional metabolic profiles [8]. The biological pattern is consistent with the personal fat threshold concept, with lean disease emerging when storage capacity is exceeded at lower body weight.

The hypertriglyceridemic waist, originally defined in cardiometabolic populations as the combination of elevated waist circumference and elevated fasting triglycerides, has been linked to NAFLD/MASLD and to visceral adiposity in subsequent cohorts [9]. It is not a MASLD-specific phenotype, but it combines two routinely available measurements (waist circumference and fasting triglycerides) and is useful as a low-resource bedside indicator of adipose-related risk. It does not distinguish VAT quantity from adipose functional state.

### 4.5. Reconciling Findings Across Methods

Method choice explains much of the variability in published associations between body composition and MASLD outcomes. Cohorts using BIA-based, mass-only sarcopenia definitions have shown weaker and more variable associations than cohorts using CT-defined muscle quality [75,76,77]. Cohorts using surrogate VAT indices generally report smaller or less specific effects than cohorts using CT/MRI VAT, because surrogates combine quantity and function [9,31,32]. Studies that include functional adipose markers such as Adipo-IR, adiponectin, or SAT fibrogenesis often show stronger links to histology than studies measuring depot quantity alone [11,22,29,90].

Several caveats apply. Most cited cohorts are cross-sectional, limiting causal inference. Sex differences are also incompletely captured: many cited cohorts do not stratify by sex, and the data that exist suggest meaningful sex modification of muscle-to-fat ratios, adipokine profiles, and disease progression [74]. Sex-stratified analyses are an active area in current MASLD body-composition work. Reverse causation is especially important in advanced disease: sarcopenia in decompensated cirrhosis ranges from 40% to 70% depending on definition and is intertwined with malnutrition, hospitalization, ascites, and fluid shifts [91]. Anthropometric and BIA measurements become unreliable in this setting, and frailty rather than mass-based sarcopenia may best capture functional reserve in NASH cirrhosis on the transplant waitlist (HR 2.36, 95% CI 1.23–4.52) [92,93]. Body-composition phenotyping is most informative in non-cirrhotic and compensated MASLD/MASH; in advanced disease, body composition increasingly reflects functional reserve rather than upstream adipose biology.

Sex hormones influence adipose depot expandability: estrogen supports subcutaneous depot expansion, whereas the postmenopausal state and androgen milieu favor visceral accumulation [94]. In MASLD cohorts, premenopausal women typically show lower visceral adipose tissue burden and lower fibrosis prevalence than men matched for body mass index, and this relative protection is attenuated after menopause. Muscle-to-visceral-fat ratios and adipokine profiles also stratify differently by sex [66], with adiponectin generally higher in women and adipose tissue insulin resistance showing sex-modified associations with hepatic histology.

### 4.6. Population Differences and the Need for Calibrated Cutpoints

Body-composition thresholds developed in Western cohorts translate imperfectly across populations. Lean MASLD is markedly more prevalent in East Asian cohorts than in Western cohorts at a comparable body mass index, consistent with earlier crossing of the personal fat threshold in populations with lower baseline subcutaneous depot capacity. The Asian Working Group for Sarcopenia has published Asian-specific muscle-mass and function cutpoints that differ from European Working Group values [62], and CT- and MRI-defined visceral-fat thresholds derived from European or North American cohorts underdiagnose visceral adiposity when applied to South and East Asian populations at equivalent metabolic risk. Universally calibrated or population-specific body-composition cutpoints for MASLD risk stratification represent an active area of methodologic work and an unresolved practical need for clinical implementation.

## 5. Therapeutic Developments in MASH and Adipose-Liver Mechanisms

**Figure 3:**
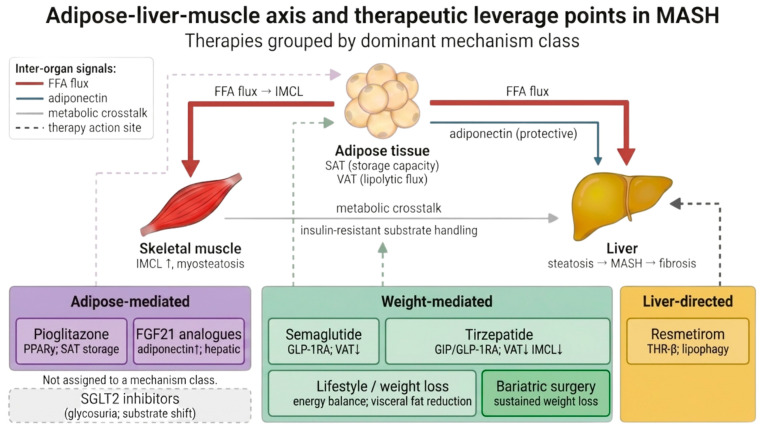
Adipose–liver–muscle axis and therapeutic leverage points in MASLD/MASH. Adipose dysfunction can present as limited SAT expandability or fibrogenesis, VAT lipolysis and FFA flux, Adipo-IR, altered adipokines, and muscle lipid infiltration. Solid arrows denote inter-organ signals—free fatty acid (FFA) flux (red), adiponectin (blue), and metabolic crosstalk (gray); dashed arrows mark sites of therapeutic action. Therapies cluster into three mechanism classes (color-coded): adipose-mediated (pioglitazone, FGF21 analogs), weight-mediated (incretin therapies, bariatric surgery, lifestyle interventions), and liver-directed (resmetirom). Sodium-glucose cotransporter 2 (SGLT2) inhibitors are shown separately in gray because their dominant actions, glycosuria and a shift toward fatty-substrate use do not map onto these three classes. Within these classes, pioglitazone primarily modifies adipose biology, FGF21 analogues target adipose tissue and reduce hepatic fibrosis, bariatric surgery produces the largest histologic effect through sustained large-scale weight loss, incretin therapies mainly reduce body weight and visceral/ectopic fat, SGLT2 inhibitors alter substrate handling in small imaging studies, lifestyle interventions reduce visceral adiposity and hepatic fat, and resmetirom acts mainly through hepatic thyroid hormone receptor beta (THR-β) pathways [21,29,30,95,97,99,100,103,104,105]. Created in BioRender. Cruz, A. J. (2026) https://BioRender.com/1tqdxp6 (accessed on 25 July 2026).

MASH therapeutics changed substantially in 2024 and 2025: liver-directed pharmacotherapy and incretin-based therapies became clinically available (Figure 3). Resmetirom received accelerated FDA approval in 2024 for adults with non-cirrhotic NASH with moderate-to-advanced fibrosis, alongside diet and exercise [95,96]. Phase 3 results for semaglutide in MASH were published in 2025, and FDA approval of Wegovy for non-cirrhotic MASH with moderate-to-advanced fibrosis followed in August 2025 [97,98]. Tirzepatide phase 2 data showed MASH resolution in 44–62% of treated patients and fibrosis improvement in roughly half at 52 weeks [99]. A network meta-analysis of 29 RCTs (n = 9324) ranked tirzepatide, resmetirom, and semaglutide as significantly better than placebo for fibrosis regression, with pegozafermin and survodutide ranking highly for MASH resolution alongside tirzepatide [100]. A 2025 conference abstract also reported pooled glucagon-like peptide 1 (GLP-1) receptor agonist effects on MASH resolution and fibrosis improvement [101]. Among weight-mediated interventions, bariatric surgery has produced the largest reported histologic effect in MASH: in a prospective 5-year follow-up by Lassailly and colleagues, NASH resolved without worsening fibrosis in 84% of patients with paired biopsies and fibrosis improved in 70% [102]. The magnitude likely reflects sustained large-scale weight loss rather than a direct adipose-targeted mechanism, and represents the practical upper limit of weight-mediated approaches in MASH. Where these agents act differs by mechanism: some work primarily on adipose biology, others through weight loss and visceral-fat reduction, and others through direct hepatic pathways. Table 3 summarizes the mechanisms and adipose-related signals of the major MASH therapies discussed in this section.

### 5.1. Adipose-Related Therapies

Pioglitazone, a peroxisome proliferator-activated receptor (PPAR)-γ agonist, has the longest history among MASH therapies that act substantially through adipose tissue. In the Belfort trial, 55 patients with biopsy-proven NASH and impaired glucose tolerance or type 2 diabetes received pioglitazone or placebo for 6 months on a hypocaloric diet. Pioglitazone reduced hepatic fat by MR spectroscopy by 54% (vs. 0% with placebo, *p* < 0.001), increased hepatic insulin sensitivity by 48% (vs. 14%, *p* = 0.008), and improved steatosis, ballooning, and inflammation, although fibrosis change did not reach significance (*p* = 0.08) [106]. The 18-month randomized trial by Cusi and colleagues in 101 patients with NASH and prediabetes or type 2 diabetes extended these findings. At 18 months, 58% achieved the primary histologic outcome and 51% achieved NASH resolution, with histologic and metabolic improvements sustained in patients who continued therapy through an open-label extension [103]. A meta-analysis of randomized trials reported that thiazolidinedione therapy, especially pioglitazone, was associated with improvement in advanced fibrosis and NASH resolution [107]. The PIVENS follow-up showed that pioglitazone produced an early reduction in Adipo-IR at 16 weeks that was not sustained at 96 weeks, whereas vitamin E improved liver histology without changing Adipo-IR. Adipo-IR change at 96 weeks was nonetheless associated with histologic improvement including fibrosis [44]. Pioglitazone’s clinical limitations—weight gain, fluid retention, and fracture risk—continue to restrict broad use.

Peroxisome proliferator-activated receptor gamma (PPARγ) is a nuclear receptor that heterodimerizes with RXR and binds peroxisome proliferator response elements in target-gene promoters. Tontonoz and Spiegelman characterized PPARγ as a central regulator of adipocyte differentiation and the principal pharmacologic target of thiazolidinediones [108]. Lehmann and colleagues demonstrated that thiazolidinediones bind PPARγ with nanomolar affinity, establishing the receptor as the molecular target for the class [109]. PPARγ target genes in adipose tissue include ADIPOQ, SLC2A4, LPL, FABP4, PLIN1, and CD36 [108]. Their coordinated induction promotes adipocyte differentiation, expands SAT triglyceride storage capacity, and increases adiponectin secretion. The clinical signature—reduced hepatic triglyceride content, improved adipose insulin sensitivity, and increased adiponectin—links pioglitazone’s adipose effects to hepatic benefit through the AdipoR2-AMPK pathway described in Section 3.4. The persistence of histologic benefit despite only transient Adipo-IR improvement suggests that Adipo-IR captures one functional axis, not the full set of adipose changes induced by PPARγ activation [44,103].

FGF21 analogs constitute a second class of agents with substantial adipose action. Native fibroblast growth factor 21 was characterized by Kharitonenkov and colleagues as a hepatokine that increased GLUT1 expression and insulin-independent glucose uptake in adipocytes [110]. Subsequent work by Badman and colleagues placed FGF21 in the peroxisome proliferator-activated receptor alpha (PPARα)-driven hepatic response to fasting [111]. FGF21 signals through FGF receptor 1c, with FGFR2c and FGFR3c also responsive in some tissues, in complex with the obligate co-receptor beta-klotho (β-klotho) [112]. BonDurant and colleagues subsequently demonstrated using adipose-specific β-klotho deletion that direct adipose signaling is required for FGF21’s acute insulin-sensitizing effect [113]. In adipose tissue, Lin and colleagues showed that FGF21 induces ADIPOQ expression in a PPARγ-dependent manner [114], and Fisher and colleagues demonstrated that FGF21 activates a PGC-1α-dependent thermogenic program in white adipose tissue [115]. Holland and colleagues separately showed that the metabolic benefit of FGF21 in obese mice required adiponectin, defining an FGF21-adiponectin-ceramide axis [116]. Hepatic effects of FGF21 include reduced SREBP1c-driven de novo lipogenesis, increased fatty-acid oxidation, and ketogenesis [111,112]. Two long-acting FGF21 analogues have phase 2b data in MASH. Harrison and colleagues randomized 128 patients with biopsy-proven MASH and F2 or F3 fibrosis to once-weekly subcutaneous efruxifermin (28 mg or 50 mg) or placebo in HARMONY. At 24 weeks, fibrosis improvement by at least one stage without worsening of MASH occurred in 39% (28 mg) and 41% (50 mg) versus 20% with placebo. At 96 weeks, response rates were 46% (28 mg) and 75% (50 mg) versus 24% with placebo, with concurrent reductions in liver fat, transaminases, and noninvasive fibrosis markers [117,118]. Loomba and colleagues reported in a separate phase 2b trial that pegozafermin given subcutaneously at 30 mg weekly or 44 mg every 2 weeks for 24 weeks improved fibrosis and reduced liver fat in patients with biopsy-proven MASH and F2 or F3 fibrosis [104].

GLP-1 receptor agonists are now central to MASH therapy, but their adipose-related effects are primarily weight-mediated. In a phase 2 trial of 320 patients, semaglutide produced NASH resolution in 59% of the highest-dose 0.4 mg daily group versus 17% with placebo at 72 weeks (*p* < 0.001), although fibrosis improvement was not statistically significant (43% vs. 33%, *p* = 0.48) [119]. The phase 3 ESSENCE trial randomized 1197 patients with biopsy-proven MASH and F2-F3 fibrosis 2:1 to once-weekly semaglutide 2.4 mg or placebo for 240 weeks. In the planned week-72 interim analysis of the first 800 randomized patients, semaglutide met both primary histologic endpoints: MASH resolution without worsening of fibrosis in 62.9% versus 34.3% and fibrosis improvement without worsening of MASH in 36.8% versus 22.4% [97]. Tirzepatide, a dual glucose-dependent insulinotropic polypeptide (GIP)/GLP-1 receptor agonist, achieved MASH resolution without worsening of fibrosis in 44%, 56%, and 62% of patients receiving 5, 10, and 15 mg weekly, compared with 10% on placebo; fibrosis improvement by at least one stage occurred in 55%, 51%, and 51% versus 30% on placebo in SYNERGY-NASH [99]. In an exploratory analysis of SYNERGY-NASH completers, MASH-resolution responders showed greater improvements than nonresponders in body weight, HbA1c, MRI-PDFF liver fat, Adipo-IR, and adiponectin. Fibrosis-improvement responders separated from nonresponders mainly by body weight and HbA1c, with smaller differences in adipose-function markers [120].

The GLP-1 receptor is a class B G-protein-coupled receptor expressed on pancreatic β-cells, hypothalamic neurons, enteroendocrine cells, and at lower levels in heart and kidney. Drucker has reviewed its signaling architecture, including Gαs/cAMP signaling through PKA and Epac [121]. Direct functional GLP-1 receptor expression in human adipocytes remains contested, so the adipose-related effects of GLP-1 receptor agonists—higher adiponectin and improved Adipo-IR—are best interpreted mainly as downstream consequences of weight loss, reduced visceral fat, and improved glycemia rather than direct adipocyte receptor signaling [121]. Across incretin trials, MASH resolution has generally exceeded fibrosis improvement at the same timepoint, in keeping with a mechanism that rapidly reduces steatosis and inflammatory activity, while fibrosis remodeling proceeds more slowly [97,100,120].

Laboratory evidence on direct adipocyte signaling remains mixed. Some studies have detected low-level GLP-1 receptor transcript in human adipose tissue and reported effects of GLP-1 receptor agonists on adipocyte lipolysis, adipogenesis, and inflammation in ex vivo systems [122], whereas other studies using highly specific antibodies and functional assays have found no evidence of direct signaling [123].

SGLT2 inhibitors act through a different metabolic pathway. Ferrannini and colleagues demonstrated that SGLT2 inhibition shifts whole-body fuel use toward fatty substrates, with elevated fasting β-hydroxybutyrate and a lower insulin-to-glucagon ratio reflecting a mild ketogenic state [105]. Small MASLD/NAFLD imaging studies suggest that hepatic fat and visceral fat can decrease during SGLT2 inhibitor therapy, but histologic MASH evidence and adipose-function biomarkers remain limited [124]. Metformin has been reported to reduce visceral fat volume through fatty-acid oxidation mechanisms in human and rodent studies, although it is not used as a MASH-specific therapy [125].

Lifestyle and nutritional interventions remain foundational in MASLD management. Sustained energy restriction, Mediterranean-style dietary patterns, reduction in ultra-processed food intake, and structured physical activity produce reductions in hepatic steatosis, and improvements in adipose tissue insulin resistance and adiponectin. At sustained weight loss of at least 5–10%, they produce meaningful histologic improvement in MASH [126]. Recent MASLD guidelines from multiple societies converge on these lifestyle recommendations as first-line therapy, both preceding and alongside pharmacotherapy [4,5]. Structured aerobic and resistance exercise programs also protect muscle mass and thereby address one of the axes discussed in Section 4. Pharmacotherapies discussed in the following sections should be considered in the context of, rather than as substitutes for, lifestyle-based approaches.

### 5.2. Resmetirom

Resmetirom is a thyroid hormone receptor-β (THR-β) agonist with selectivity for THR-β over THR-α. In the phase 3 MAESTRO-NASH trial, resmetirom met both primary histologic endpoints at 52 weeks. Among 966 patients with biopsy-proven MASH and F1B-F3 fibrosis, NASH resolution without worsening of fibrosis occurred in 25.9% of patients receiving 80 mg and 29.9% receiving 100 mg versus 9.7% with placebo. Fibrosis improvement by at least one stage without worsening of NAFLD activity score occurred in 24.2% and 25.9% versus 14.2%, respectively (*p* < 0.001 for both dose comparisons with placebo for both endpoints) [95]. Body-weight reduction was modest overall. The trial also reported MRI-PDFF response, defined as a relative reduction of at least 30% in liver fat, as a key noninvasive endpoint that has subsequently been used to gauge hepatic fat response [95].

Two MAESTRO-NASH observations clarify the relationship between weight loss and histologic response. In a secondary analysis, concurrent weight loss was associated with higher response rates. Among resmetirom-treated patients with at least 5% weight loss, 90.9% in the 80 mg group and 97.7% in the 100 mg group achieved at least 30% MRI-PDFF reduction. In the 100 mg group, MASH resolution was 56.6% with at least 5% weight loss versus 33.8% with <5% weight loss, and fibrosis improvement was 40.6% versus 31.5% [127]. Patients receiving stable background GLP-1 receptor agonist or SGLT2 inhibitor therapy had response rates similar to those not receiving these therapies [127].

THR-β is the predominant thyroid hormone receptor isoform in liver. THR-β heterodimerizes with RXR and binds thyroid hormone response elements in target genes that include DIO1, ME1, ANGPTL4, CYP7A1, and LDLR, alongside genes encoding components of mitochondrial fatty-acid β-oxidation and the autophagy machinery [128]. THR-β-driven mitochondrial biogenesis proceeds through induction of PGC-1α and nuclear respiratory factors, and lipophagy is engaged through a CAMKK2-AMPK-ULK1 cascade that delivers triglyceride-laden lipid droplets to lysosomes [128]. De novo lipogenesis is reduced through indirect effects on SREBP1c [128]. Preclinical data support an antifibrotic component: in a mouse NASH model with advanced fibrosis, Kannt and colleagues reported that resmetirom reduced fibrosis stage and stellate-cell activation independent of body-weight change [129]. The reported 28-fold THR-β over THR-α selectivity of resmetirom in vitro [130] is intended to limit off-target effects in tissues where THR-α predominates. This liver-directed mechanism is consistent with the fibrosis benefit seen at modest weight change in MAESTRO-NASH, and indicates that adipose-focused measures are unlikely to substitute for histologic or liver-imaging endpoints in trials of THR-β agonism.

**Table 3:** Therapeutic mechanisms and adipose-related signals in MASH trials and related human studies.

Therapy/Class	Key Evidence	Dominant Mechanistic Axis	Adipose or Body-Composition Signal	Main Limitation for Interpretation
Pioglitazone/PPARγ agonist	Belfort trial [106]; Cusi long-term trial [103]; TZD meta-analysis [107]; PIVENS follow-up [44]	Adipose-directed: SAT storage, adipocyte differentiation, adiponectin; secondary hepatic benefit	Improved hepatic fat and insulin sensitivity; early Adipo-IR improvement; weight gain may occur	Side effects limit use; Adipo-IR captures only one axis of response
FGF21 analogs (efruxifermin, pegozafermin)	HARMONY phase 2b at 24 wk and 96 wk [117,118]; pegozafermin phase 2b [104]; FGF21 mechanism review [112]	Combined adipose and liver: increased adiponectin, improved insulin sensitivity, reduced hepatic DNL, antifibrotic signaling	Histologic fibrosis improvement; increased adiponectin; reduced liver fat	Phase 2b only; phase 3 results pending
Semaglutide/GLP-1RA	Phase 2 NASH trial [119]; phase 3 ESSENCE [97]; FDA approval [98]	Weight-mediated reduction in visceral and ectopic fat; improved glycemia	Large weight reduction; MRI-PDFF and Adipo-IR/adiponectin changes in responder analyses	Fibrosis response slower and smaller than MASH resolution at same timepoint
Tirzepatide/GIP-GLP-1RA	SYNERGY-NASH phase 2 [99]; participant-level exploratory analysis [120]	Weight-mediated and metabolic; larger adipose-related shifts in MASH-resolution responders	Greater weight, HbA1c, MRI-PDFF, Adipo-IR, and adiponectin changes among MASH-resolution responders	Exploratory responder analysis; phase 2 sample size
SGLT2 inhibitors	Fuel-substrate physiology [105]; small canagliflozin NAFLD imaging study [124]	Substrate shift and glycosuria; possible hepatic fat reduction	Small imaging studies show liver fat and VAT changes; adipose-function signals limited	No large histologic MASH trial signal established
Resmetirom/THR-β agonist	MAESTRO-NASH phase 3 [95]; secondary analysis [127]; FDA approval [96]	Liver-directed: THR-β effects on lipid handling, mitochondrial programs, lipophagy; preclinical antifibrotic signal	Modest weight change; response enhanced with concurrent weight loss but not dependent on it	Combination benefit with incretin/SGLT2 therapy not yet formally tested
Bariatric surgery	Lassailly 5-year follow-up [102]	Weight-mediated (sustained large-scale weight loss); produces the largest reported histologic effect among weight-mediated interventions	NASH resolution in 84% and fibrosis improvement in 70% at 5 years	Procedural and selection limitations; not pharmacologic
Lifestyle and weight loss	Longitudinal body-composition meta-analysis [131]; living donor paired CT/biopsy study [132]; lifestyle RCT [126]	Adipose and energy-balance intervention; VAT and hepatic fat reduction	Visceral-fat reduction predicts fatty-liver resolution in paired donor data	Durability and adherence vary; histologic fibrosis endpoints less consistently available

Abbreviations: FGF21, fibroblast growth factor 21; GLP-1RA, glucagon-like peptide-1 receptor agonist; GIP, glucose-dependent insulinotropic polypeptide; MRI-PDFF, magnetic resonance imaging-proton density fat fraction; PPARγ, peroxisome proliferator-activated receptor γ; SAT, subcutaneous adipose tissue; THR-β, thyroid hormone receptor β; VAT, visceral adipose tissue.

### 5.3. Body Composition and Fibrosis Response Across Recent Trials

Body-composition change and hepatic histologic change have correlated imperfectly across recent trials. Longitudinal data indicate that improved body composition can reduce hepatic fat content [131], but compartment-specific change and histologic fibrosis response do not map one-to-one. In MAESTRO-NASH, resmetirom improved fibrosis despite modest weight change, and effects were preserved in patients on stable background GLP-1 receptor agonist or SGLT2 inhibitor therapy [95,127]. Semaglutide produced substantial weight loss and MASH resolution at 72 weeks, with a smaller but significant fibrosis benefit at the same timepoint [97]. In SYNERGY-NASH, MASH-resolution responders had large improvements in body weight, HbA1c, MRI-PDFF, Adipo-IR, and adiponectin, whereas fibrosis-improvement responders differed more clearly by body weight and HbA1c than by adipose-function measures [120].

Lifestyle and observational intervention data reinforce the compartment-specific point. Lee and colleagues studied 115 potential living liver donors with NAFL and paired biopsy and CT before and after lifestyle intervention; relative reduction in visceral fat area independently predicted fatty-liver resolution, whereas relative reductions in body weight, BMI, or skeletal muscle area did not [132]. A randomized trial of therapeutic lifestyle changes in patients with overweight or obesity and NAFLD produced reductions in steatosis through diet and physical activity [126]. Conversely, a small prospective canagliflozin study (n = 9) in patients with type 2 diabetes and NAFLD showed MRI-PDFF reduction without significant correlations between PDFF change and changes in visceral fat, subcutaneous fat, or skeletal muscle area [124]. Body-composition endpoints add information that hepatic histology and liver-specific imaging cannot provide on their own, but they do not substitute for them.

### 5.4. Considerations for Measurement in MASH Trials

Adipo-IR has been used in MASLD-specific cohorts as a measure of failure of insulin to suppress adipose lipolysis, calculable from fasting NEFA and insulin. Reported associations include histologic severity in cross-sectional cohorts and differential response patterns to MASH-active interventions in trial analyses [29,44,45]. Imaging-defined VAT, when paired with hepatic imaging endpoints, has distinguished depot-specific change from overall mass loss in observational and interventional cohorts [124,132]. Imaging-defined muscle quality, including myosteatosis and NAMA/TAMA indices, measures ectopic muscle lipid that BIA-based muscle-mass indices do not capture [57,58,77].

Among clinical phenotypes, lean MASLD cohorts often show elevated VAT, Adipo-IR, and reduced adiponectin despite normal BMI [6,7,8,11]. Sarcopenic visceral obesity has been associated with advanced fibrosis and higher cardiometabolic risk in the cohorts where it has been measured, although diagnostic criteria remain unsettled [59,60,87,88]. In MAESTRO-NASH, resmetirom improved histology and fibrosis at modest overall weight change [95]; a secondary analysis reported that response was enhanced, but not dependent on, concurrent weight loss of at least 5% [127].

The mechanistic complementarity between adipose-mediated and liver-directed therapies provides a rationale for combination approaches that has not yet been formally tested in MASH. Incretin-based therapies act upstream by reducing visceral adipose tissue lipolytic flux, improving adipose tissue insulin resistance, and restoring adiponectin signaling; these effects should reduce the free fatty acid load reaching the liver. Thyroid hormone receptor β (THR-β) agonists such as resmetirom act downstream by enhancing hepatic lipid oxidation and lipophagy in hepatocytes. A combination of an upstream flux-reducing agent with a downstream liver-directed agent could plausibly achieve fibrosis regression greater than either alone, particularly in patients with concurrent metabolic disease. The MAESTRO-NASH secondary analysis showing preserved resmetirom effects on background GLP-1 receptor agonist and SGLT2 inhibitor therapy is compatible with this hypothesis but does not establish it [127]. Formal randomized combination trials are needed.

## 6. Conclusions

Clinically, adipose dysfunction presents through phenotypes such as lean MASLD, sarcopenic visceral obesity, myosteatosis, and the hypertriglyceridemic waist. Direct VAT imaging and CT/MRI-defined muscle quality provide more granular information than BMI, BIA-based muscle mass alone, or surrogate indices. Measurement of adipose tissue dysfunction through direct depot imaging, SAT fibrogenesis, Adipo-IR, and adipokines captures distinct biology: lipid storage location, extracellular-matrix remodeling, failure of insulin-mediated lipolytic suppression, and endocrine signaling. In advanced cirrhosis, however, body composition often reflects frailty and functional reserve rather than upstream adipose biology, so interpretation must shift accordingly.

Recent MASH trials show why mechanism classes must be distinguished. Pioglitazone and FGF21 analogs act substantially through adipose-mediated pathways. Incretin therapies, by contrast, work primarily through weight-mediated reduction in visceral and ectopic fat. SGLT2 inhibitors alter substrate handling, though histologic evidence remains limited. Resmetirom acts through liver-directed hepatic THR-β pathways and improves fibrosis without major weight loss. Bariatric surgery, the most studied of the weight-mediated interventions, has produced the largest histologic benefit reported to date. Secondary analyses suggest that adipose-mediated and liver-directed mechanisms may act in parallel, but formal combination trials have not yet established additive efficacy.

Integration of adipose function, fat distribution, muscle quality, and liver-specific endpoints is an active direction in current MASLD phenotyping. Such integration is not expected to replace histology or validated liver imaging, but it has been used in recent cohorts and trial analyses to refine risk stratification and therapeutic interpretation.

## Figures and Tables

**Table 1:** Methods and biomarkers used to characterize adipose-related phenotypes in MASLD studies, with main interpretive limitations.

Method	What It Captures	Strengths	Main Limitations	Best Current Use
CT	VAT/SAT area; muscle area; muscle attenuation as a myosteatosis proxy	Direct compartmental measurement; opportunistic use of routine scans; AI-enabled pipelines [55,56,57]	Radiation; protocol heterogeneity; cutpoints vary by level and population	High-value phenotyping when scans already exist
MRI	VAT/SAT volume; muscle size; muscle fat infiltration; hepatic fat by MRI-PDFF	Direct quantification without radiation; strongest for ectopic fat characterization [11]	Cost; access; analytic burden	Research-grade phenotyping and longitudinal studies
DXA	Appendicular lean mass; total and regional fat mass	Widely available; standardized lean-mass estimates [58,59]	Does not segment VAT with CT/MRI precision; limited for muscle quality	Cohorts focused on lean mass or sarcopenic obesity rather than depot distribution
BIA	Estimated appendicular muscle mass and, in some devices, visceral fat area	Fast, inexpensive, scalable; common in screening cohorts [60]	Hydration-dependent; equation-based estimates; less precise for VAT and muscle quality	Large epidemiologic studies using ratio metrics
Surrogate indices (VAI and related waist–lipid indices)	Indirect estimate of visceral adiposity or adipose dysfunction	Easily obtained without imaging [9,32]	Cannot separate VAT quantity from biology; lower specificity	Interim risk context when imaging is unavailable
Adipo-IR (NEFA × insulin)	Functional adipose insulin resistance; failure of insulin to suppress lipolysis	Two fasting measurements; tracks histology in MASLD cohorts [29,44,45]	NEFA is not routinely available in many clinics; cutpoints vary across assays and cohorts	Functional adipose axis described in Section 3.3 and useful for mechanistic trial interpretation

Abbreviations: BIA, bioelectrical impedance analysis; CT, computed tomography; DXA, dual-energy X-ray absorptiometry; MRI, magnetic resonance imaging; NEFA, non-esterified fatty acids; SAT, subcutaneous adipose tissue; VAI, visceral adiposity index; VAT, visceral adipose tissue.

**Table 2:** Representative studies linking adipose-related body-composition phenotypes to MASLD outcomes.

Phenotype Category	Study	Setting/Measurement	Main Finding	Main Limitation
Depot quantity (VAT)	Yu 2015 [31]	Biopsy-proven NAFLD, n = 324; CT VAT area	VAT increased stepwise across controls/NAFLD/fibrosis groups; 1-SD VAT increase doubled odds of significant fibrosis	Cross-sectional; no muscle phenotyping
Depot quantity and dysfunction	Saponaro 2022 [11]	Lean/non-lean NAFLD; biopsy + MRI + tracer, n = 32 NAFLD + 8 controls	VAT elevated even in lean NAFLD; VAT and palmitate flux tracked multi-tissue IR, reduced adiponectin, and NASH	Selected cohort; combines depot quantity and dysfunction
Depot fibrogenesis	Beals 2021 [21]	In vivo D2O labeling of SAT; n = 12 lean, 24 obese non-NAFLD, 25 obese NAFLD	SAT lipogenic capacity preserved; SAT collagen synthesis elevated in obese NAFLD	Single study; SAT only; not validated as biomarker
Depot fibrogenesis	Bilson 2024 [22]	NAFLD cohort, n = 62; SAT biopsy + VCTE	SAT composite collagen gene expression associated with clinically significant fibrosis	Modest n; cross-sectional; biopsy-based assay
Adipo-IR	Lomonaco 2012 [29]	MR spectroscopy + euglycemic clamp ± biopsy; n = 207 NAFLD	Adipo-IR elevated in NAFLD and tracked hepatic IR, IHTG, inflammation, ballooning, and fibrosis stage	Cross-sectional; biopsy subset
Adipo-IR	Kalavalapalli 2023 [45]	T2D + NAFLD cohort; VCTE-defined fibrosis	Adipo-IR independently predicted clinically significant fibrosis; HOMA-IR and CK-18 lost significance after adjustment	VCTE rather than biopsy; T2D-enriched
Skeletal muscle quality	Hsieh 2023 [58]	Early-stage NAFLD; CT-defined myosteatosis; longitudinal subset	Severe myosteatosis, not mass-based sarcopenia, associated with early NASH and fibrosis progression	Longitudinal subset modest
Skeletal muscle quality	Nachit 2023 [57]	Asymptomatic adults; AI-CT body composition; mortality endpoint	CT myosteatosis independently predicted all-cause mortality	General prognostic context; not liver-specific
Composite phenotype	Kim 2023 [59]	Non-obese NAFLD; visceral fat obesity + sarcopenia + myosteatosis	Combined adverse phenotype identified higher-risk non-obese disease	Cross-sectional; non-obese focus
Composite phenotype	Elsabaawy 2025 [60]	MASLD cohort, n = 334; MRI VAT + BIA sarcopenia + MRE	SVO present in 42.5%; independently predicted advanced fibrosis; risk tiers proposed	Single cohort; SVO criteria not standardized
Lean disease	Cheah 2025 [8]	Lean-MAFLD meta-analysis; >10 million individuals	Lean-MAFLD associated with higher liver-related mortality than overweight/obese MAFLD	MAFLD-specific definition; high heterogeneity

Abbreviations: ASCVD, atherosclerotic cardiovascular disease; BIA, bioelectrical impedance analysis; CT, computed tomography; D2O, deuterated water; DXA, dual-energy X-ray absorptiometry; IHTG, intrahepatic triglyceride; IR, insulin resistance; MAFLD, metabolic dysfunction-associated fatty liver disease; MRE, magnetic resonance elastography; NAMA, normal-attenuation muscle area; SAT, subcutaneous adipose tissue; SO, sarcopenic obesity; SVO, sarcopenic visceral obesity; T2D, type 2 diabetes; TAMA, total abdominal muscle area; VAT, visceral adipose tissue; VCTE, vibration-controlled transient elastography. Legacy NAFLD/NASH/MAFLD terminology is retained where used by the original study.

## Data Availability

No new data were created in this study. Data sharing is not applicable.

## References

[1] Younossi Z.M., Golabi P., Paik J.M., Henry A., Van Dongen C., Henry L. (2023). The global epidemiology of nonalcoholic fatty liver disease (NAFLD) and nonalcoholic steatohepatitis (NASH): A systematic review. Hepatology.

[2] Riazi K., Azhari H., Charette J.H., Underwood F.E., King J.A., Afshar E.E., Swain M.G., Congly S.E., Kaplan G.G., Shaheen A.A. (2022). The prevalence and incidence of NAFLD worldwide: A systematic review and meta-analysis. Lancet Gastroenterol. Hepatol..

[3] Rinella M.E., Lazarus J.V., Ratziu V., Francque S.M., Sanyal A.J., Kanwal F., Romero D., Abdelmalek M.F., Anstee Q.M., Arab J.P. (2023). A multisociety Delphi consensus statement on new fatty liver disease nomenclature. J. Hepatol..

[4] Rinella M.E., Neuschwander-Tetri B.A., Siddiqui M.S., Abdelmalek M.F., Caldwell S., Barb D., Kleiner D.E., Loomba R. (2023). AASLD Practice Guidance on the clinical assessment and management of nonalcoholic fatty liver disease. Hepatology.

[5] Cusi K., Abdelmalek M.F., Apovian C.M., Balapattabi K., Bannuru R.R., Barb D., Bardsley J.K., Beverly E.A., Corbin K.D., ElSayed N.A. (2025). Metabolic dysfunction-associated steatotic liver disease (MASLD) in people with diabetes: The need for screening and early intervention. A consensus report of the American Diabetes Association. Diabetes Care.

[6] Stefan N., Yki-Järvinen H., Neuschwander-Tetri B.A. (2025). Metabolic dysfunction-associated steatotic liver disease: Heterogeneous pathomechanisms and effectiveness of metabolism-based treatment. Lancet Diabetes Endocrinol..

[7] Wakabayashi S.-I., Tamaki N., Kimura T., Umemura T., Kurosaki M., Izumi N. (2024). Natural history of lean and non-lean metabolic dysfunction-associated steatotic liver disease. J. Gastroenterol..

[8] Cheah M.C.C., Crane H., George J. (2025). Global prevalence, metabolic characteristics, and outcomes of lean-MAFLD: A systematic review and meta-analysis. Hepatol. Int..

[9] Hanlon C.L., Yuan L. (2022). Nonalcoholic Fatty Liver Disease: The role of visceral adipose tissue. Clin. Liver Dis..

[10] Wang X., Rao H.Y., Liu F., Wei L., Li H., Wu C. (2021). Recent advances in adipose tissue dysfunction and its role in the pathogenesis of non-alcoholic fatty liver disease. Cells.

[11] Saponaro C., Sabatini S., Gaggini M., Carli F., Rosso C., Positano V., Armandi A., Caviglia G.P., Faletti R., Bugianesi E. (2022). Adipose tissue dysfunction and visceral fat are associated with hepatic insulin resistance and severity of NASH even in lean individuals. Liver Int..

[12] Bansal S.K., Bansal M.B. (2024). Pathogenesis of MASLD and MASH—Role of insulin resistance and lipotoxicity. Aliment. Pharmacol. Ther..

[13] Targher G., Byrne C.D., Tilg H. (2024). MASLD: A systemic metabolic disorder with cardiovascular and malignant complications. Gut.

[14] Taylor R., Holman R.R. (2015). Normal weight individuals who develop type 2 diabetes: The personal fat threshold. Clin. Sci..

[15] Marjot T., Armstrong M.J., Stine J.G. (2025). Skeletal muscle and MASLD: Mechanistic and clinical insights. Hepatol. Commun..

[16] Henin G., Loumaye A., Leclercq I.A., Lanthier N. (2024). Myosteatosis: Diagnosis, pathophysiology and consequences in metabolic dysfunction-associated steatotic liver disease. JHEP Rep..

[17] Barbalho S.M., Laurindo L.F., Valenti V.E., Méndez-Sánchez N., Ramírez-Mejía M.M., de Alvares Goulart R. (2025). Organokine-mediated crosstalk: A systems biology perspective on the pathogenesis of MASLD—A narrative review. Int. J. Mol. Sci..

[18] Sandireddy R., Sakthivel S., Gupta P., Behari J., Tripathi M., Singh B.K. (2024). Systemic impacts of MASLD and MASH on heart, muscle, and kidney related diseases. Front. Cell Dev. Biol..

[19] Geng Y., Faber K.N., de Meijer V.E., Blokzijl H., Moshage H. (2021). How does hepatic lipid accumulation lead to lipotoxicity in non-alcoholic fatty liver disease?. Hepatol. Int..

[20] Raverdy V., Tavaglione F., Chatelain E., Lassailly G., De Vincentis A., Vespasiani-Gentilucci U., Qadri S.F., Caiazzo R., Verkindt H., Saponaro C. (2024). Data-driven cluster analysis identifies distinct types of metabolic dysfunction-associated steatotic liver disease. Nat. Med..

[21] Beals J.W., Smith G.I., Shankaran M., Fuchs A., Schweitzer G.G., Yoshino J., Field T., Matthews M., Nyangau E., Morozov D. (2021). Increased adipose tissue fibrogenesis, not impaired expandability, is associated with nonalcoholic fatty liver disease. Hepatology.

[22] Bilson J., Oquendo C.J., Read J., Scorletti E., Afolabi P.R., Lord J., Bindels L.B., Targher G., Mahajan S., Baralle D. (2024). Markers of adipose tissue fibrogenesis associate with clinically significant liver fibrosis and are unchanged by synbiotic treatment in patients with NAFLD. Metabolism.

[23] Taylor R., Barnes A.C., Hollingsworth K.G., Irvine K.M., Solovyova A.S., Clark L., Kelly T., Martin-Ruiz C., Romeres D., Koulman A. (2023). Aetiology of type 2 diabetes in people with a ‘normal’ body mass index: Testing the personal fat threshold hypothesis. Clin. Sci..

[24] Virtue S., Vidal-Puig A. (2010). Adipose tissue expandability, lipotoxicity and the Metabolic Syndrome—An allostatic perspective. Biochim. Biophys. Acta.

[25] Trépo E., Valenti L. (2020). Update on NAFLD genetics: From new variants to the clinic. J. Hepatol..

[26] Romeo S., Kozlitina J., Xing C., Pertsemlidis A., Cox D., Pennacchio L.A., Boerwinkle E., Cohen J.C., Hobbs H.H. (2008). Genetic variation in PNPLA3 confers susceptibility to nonalcoholic fatty liver disease. Nat. Genet..

[27] He S., McPhaul C., Li J.Z., Garuti R., Kinch L., Grishin N.V., Cohen J.C., Hobbs H.H. (2010). A sequence variation (I148M) in PNPLA3 associated with nonalcoholic fatty liver disease disrupts triglyceride hydrolysis. J. Biol. Chem..

[28] BasuRay S., Smagris E., Cohen J.C., Hobbs H.H. (2017). The PNPLA3 variant associated with fatty liver disease (I148M) accumulates on lipid droplets by evading ubiquitylation. Hepatology.

[29] Lomonaco R., Ortiz-Lopez C., Orsak B., Webb A., Hardies J., Darland C., Finch J., Gastaldelli A., Harrison S., Tio F. (2012). Effect of adipose tissue insulin resistance on metabolic parameters and liver histology in obese patients with nonalcoholic fatty liver disease. Hepatology.

[30] Boutari C., Mantzoros C.S. (2020). Adiponectin and leptin in the diagnosis and therapy of NAFLD. Metabolism.

[31] Yu S.J., Kim W., Kim D., Yoon J.H., Lee K., Kim J.H., Cho E.J., Lee J.H., Kim H.Y., Kim Y.J. (2015). Visceral obesity predicts significant fibrosis in patients with nonalcoholic fatty liver disease. Medicine.

[32] Ismaiel A., Dumitrascu D.L. (2021). The visceral adiposity index in non-alcoholic fatty liver disease and liver fibrosis: A systematic review and meta-analysis. Biomedicines.

[33] Chau Y.-Y., Bandiera R., Serrels A., Martínez-Estrada O.M., Qing W., Lee M., Slight J., Thornburn A., Berry R., McHaffie S. (2014). Visceral and subcutaneous fat have different origins and evidence supports a mesothelial source. Nat. Cell Biol..

[34] Tchkonia T., Thomou T., Zhu Y., Karagiannides I., Pothoulakis C., Jensen M.D., Kirkland J.L. (2013). Mechanisms and metabolic implications of regional differences among fat depots. Cell Metab..

[35] Arner P. (1999). Catecholamine-induced lipolysis in obesity. Int. J. Obes. Relat. Metab. Disord..

[36] Item F., Konrad D. (2012). Visceral fat and metabolic inflammation: The portal theory revisited. Obes. Rev..

[37] Donnelly K.L., Smith C.I., Schwarzenberg S.J., Jessurun J., Boldt M.D., Parks E.J. (2005). Sources of fatty acids stored in liver and secreted via lipoproteins in patients with nonalcoholic fatty liver disease. J. Clin. Investig..

[38] Sun K., Kusminski C.M., Scherer P.E. (2011). Adipose tissue remodeling and obesity. J. Clin. Investig..

[39] Cinti S., Mitchell G., Barbatelli G., Murano I., Ceresi E., Faloia E., Wang S., Fortier M., Greenberg A.S., Obin M.S. (2005). Adipocyte death defines macrophage localization and function in adipose tissue of obese mice and humans. J. Lipid Res..

[40] Halberg N., Khan T., Trujillo M.E., Wernstedt-Asterholm I., Attie A.D., Sherwani S., Wang Z.V., Landskroner-Eiger S., Dineen S., Magalang U.J. (2009). Hypoxia-inducible factor 1α induces fibrosis and insulin resistance in white adipose tissue. Mol. Cell. Biol..

[41] Khan T., Muise E.S., Iyengar P., Wang Z.V., Chandalia M., Abate N., Zhang B.B., Bonaldo P., Chua S., Scherer P.E. (2009). Metabolic dysregulation and adipose tissue fibrosis: Role of collagen VI. Mol. Cell. Biol..

[42] Lomonaco R., Bril F., Portillo-Sanchez P., Ortiz-Lopez C., Orsak B., Biernacki D., Lo M., Suman A., Weber M.H., Cusi K. (2016). Metabolic impact of nonalcoholic steatohepatitis in obese patients with type 2 diabetes. Diabetes Care.

[43] Bril F., Kalavalapalli S., Lomonaco R., Frye R., Godinez Leiva E., Cusi K. (2024). Insulin resistance is an integral feature of MASLD even in the presence of PNPLA3 variants. JHEP Rep..

[44] Bell L.N., Wang J., Muralidharan S., Chalasani S., Fullenkamp A.M., Wilson L.A., Sanyal A.J., Kowdley K.V., Neuschwander-Tetri B.A., Brunt E.M. (2012). Relationship between adipose tissue insulin resistance and liver histology in NASH: A PIVENS follow-up study. Hepatology.

[45] Kalavalapalli S., Godinez Leiva E., Lomonaco R., Chi X., Shrestha S., Dillard R., Budd J., Romero J.P., Li C., Bril F. (2023). Adipose tissue insulin resistance predicts the severity of liver fibrosis in patients with type 2 diabetes and NAFLD. J. Clin. Endocrinol. Metab..

[46] Samuel V.T., Shulman G.I. (2012). Mechanisms for insulin resistance: Common threads and missing links. Cell.

[47] Petersen M.C., Shulman G.I. (2018). Mechanisms of insulin action and insulin resistance. Physiol. Rev..

[48] Straub L.G., Scherer P.E. (2019). Metabolic Messengers: Adiponectin. Nat. Metab..

[49] Yamauchi T., Nio Y., Maki T., Kobayashi M., Takazawa T., Iwabu M., Okada-Iwabu M., Kawamoto S., Kubota N., Kubota T. (2007). Targeted disruption of AdipoR1 and AdipoR2 causes abrogation of adiponectin binding and metabolic actions. Nat. Med..

[50] Hardie D.G. (2007). AMP-activated/SNF1 protein kinases: Conserved guardians of cellular energy. Nat. Rev. Mol. Cell Biol..

[51] Asahina K., Zhou B., Pu W.T., Tsukamoto H. (2011). Septum transversum-derived mesothelium gives rise to hepatic stellate cells and perivascular mesenchymal cells in developing mouse liver. Hepatology.

[52] Shrestha S., Jeon J.H., Hong C.W. (2025). Neutrophils in MASLD and MASH. BMB Rep..

[53] Xia Y., Wang Y., Xiong Q., He J., Wang H., Islam M., Zhou X., Kim A., Zhang H., Huang H. (2025). Neutrophil extracellular traps promote MASH fibrosis by metabolic reprogramming of HSC. Hepatology.

[54] Babuta M., Morel C., de Carvalho Ribeiro M., Calenda C., Ortega-Ribera M., Thevkar Nagesh P., Copeland C., Zhuang Y., Wang Y., Cho Y. (2024). Neutrophil extracellular traps activate hepatic stellate cells and monocytes via NLRP3 sensing in alcohol-induced acceleration of MASH fibrosis. Gut.

[55] Shen W., Punyanitya M., Wang Z., Gallagher D., St-Onge M.-P., Albu J., Heymsfield S.B., Heshka S. (2004). Total body skeletal muscle and adipose tissue volumes: Estimation from a single abdominal cross-sectional image. J. Appl. Physiol..

[56] Mourtzakis M., Prado C.M.M., Lieffers J.R., Reiman T., McCargar L.J., Baracos V.E. (2008). A practical and precise approach to quantification of body composition in cancer patients using computed tomography images acquired during routine care. Appl. Physiol. Nutr. Metab..

[57] Nachit M., Horsmans Y., Summers R.M., Leclercq I.A., Pickhardt P.J. (2023). AI-based CT body composition identifies myosteatosis as key mortality predictor in asymptomatic adults. Radiology.

[58] Hsieh Y.C., Joo S.K., Koo B.K., Lin H.-C., Lee D.H., Chang M.S., Park J.H., So Y.H., Kim W. (2023). Myosteatosis, but not Sarcopenia, Predisposes NAFLD Subjects to Early Steatohepatitis and Fibrosis Progression. Clin. Gastroenterol. Hepatol..

[59] Kim H.-K., Bae S.-J., Lee M.J., Kim E.H., Park H., Kim H.S., Cho Y.K., Jung C.H., Lee W.J., Choe J. (2023). Association of visceral fat obesity, sarcopenia, and myosteatosis with non-alcoholic fatty liver disease without obesity. Clin. Mol. Hepatol..

[60] Elsabaawy M., Ragab A., Abd-Elrazek A., Atef M., Naguib M. (2025). Sarcopenic visceral obesity in patients with metabolic dysfunction-associated steatotic liver disease (MASLD). Clin. Exp. Med..

[61] Zhang W., Huang R., Wang Y., Rao H., Wei L., Su G.L., Lok A.S. (2019). Fat accumulation, liver fibrosis, and metabolic abnormalities in Chinese patients with moderate/severe versus mild hepatic steatosis. Hepatol. Commun..

[62] Chen L.K., Woo J., Assantachai P., Auyeung T.W., Chou M.Y., Iijima K., Jang H.C., Kang L., Kim M., Kim S. (2020). Asian Working Group for Sarcopenia: 2019 Consensus Update on Sarcopenia Diagnosis and Treatment. J. Am. Med. Dir. Assoc..

[63] Iwaki M., Kobayashi T., Nogami A., Saito S., Nakajima A., Yoneda M. (2023). Impact of sarcopenia on non-alcoholic fatty liver disease. Nutrients.

[64] Shida T., Akiyama K., Oh S., Sawai A., Isobe T., Okamoto Y., Ishige K., Mizokami Y., Yamagata K., Onizawa K. (2018). Skeletal muscle mass to visceral fat area ratio is an important determinant affecting hepatic conditions of non-alcoholic fatty liver disease. J. Gastroenterol..

[65] Shi Y.X., Chen X.Y., Qiu H.N., Jiang W.R., Zhang M.Y., Huang Y.P., Ji Y.P., Zhang S., Li C.J., Lin J.N. (2021). Visceral fat area to appendicular muscle mass ratio as a predictor for nonalcoholic fatty liver disease independent of obesity. Scand. J. Gastroenterol..

[66] Cho Y., Chang Y., Ryu S., Jung H.-S., Kim C.-W., Oh H., Kim M.K., Sohn W., Shin H., Wild S.H. (2022). Skeletal muscle mass to visceral fat area ratio as a predictor of NAFLD in lean and overweight men and women with effect modification by sex. Hepatol. Commun..

[67] Xing M., Ni Y., Zhang Y., Zhao X., Yu X. (2023). The relationship between skeletal muscle mass to visceral fat area ratio and metabolic dysfunction-associated fatty liver disease subtypes in middle-aged and elderly population. Front. Nutr..

[68] Su X., Xu J., Zheng C. (2019). The relationship between non-alcoholic fatty liver and skeletal muscle mass to visceral fat area ratio in women with type 2 diabetes. BMC Endocr. Disord..

[69] Zhang X., He Z., Si Q., Hu X., Yang L., Gu X., Du L., Wang L., Pan L., Li Y. (2022). The association of sarcopenia and visceral obesity with lean nonalcoholic fatty liver disease in Chinese patients with type 2 diabetes mellitus. J. Diabetes Res..

[70] Liu C., Li N., Sheng D., Shao Y., Qiu L., Shen C., Liu Z. (2024). Increased visceral fat area to skeletal muscle mass ratio is positively associated with the risk of metabolic dysfunction-associated steatotic liver disease in a Chinese population. Lipids Health Dis..

[71] Mai Z., Chen Y., Mao H., Wang L. (2024). Association between the skeletal muscle mass to visceral fat area ratio and metabolic dysfunction-associated fatty liver disease: A cross-sectional study of NHANES 2017–2018. J. Diabetes.

[72] Han E., Lee Y.H., Ahn S.H., Cha B.-S., Kim S.U., Lee B.-W. (2024). Appendicular skeletal muscle mass to visceral fat area ratio predicts hepatic morbidities. Gut Liver.

[73] Kim G., Lee S.E., Lee Y.B., Jun J.E., Ahn J., Bae J.C., Jin S.-M., Hur K.Y., Jee J.H., Lee M.-K. (2018). Relationship between relative skeletal muscle mass and nonalcoholic fatty liver disease: A 7-year longitudinal study. Hepatology.

[74] Li G., Rios R.S., Wang X.X., Yu Y., Zheng K.I., Huang O.-Y., Tang L.-J., Ma H.-L., Jin Y., Targher G. (2022). Sex influences the association between appendicular skeletal muscle mass to visceral fat area ratio and non-alcoholic steatohepatitis in patients with biopsy-proven non-alcoholic fatty liver disease. Br. J. Nutr..

[75] Malik A., Javaid S., Malik M.I., Qureshi S. (2024). Relationship between sarcopenia and metabolic dysfunction-associated steatotic liver disease (MASLD): A systematic review and meta-analysis. Ann. Hepatol..

[76] Li X., He J., Sun Q. (2024). The prevalence and effects of sarcopenia in patients with metabolic dysfunction-associated steatotic liver disease (MASLD): A systematic review and meta-analysis. Clin. Nutr..

[77] Kim M.J., Cho Y.K., Kim E.H., Lee M.J., Lee W.J., Kim H.-K., Jung C.H. (2024). Association between metabolic dysfunction-associated steatotic liver disease and myosteatosis measured by computed tomography. J. Cachexia Sarcopenia Muscle.

[78] Nachit M., Lanthier N., Rodriguez J., Neyrinck A.M., Cani P.D., Bindels L.B., Hiel S., Pachikian B.D., Trefois P., Thissen J.-P. (2021). A dynamic association between myosteatosis and liver stiffness: Results from a prospective interventional study in obese patients. JHEP Rep..

[79] Emanuelsson E.B., Berry D.B., Reitzner S.M., Arif M., Mardinoglu A., Gustafsson T., Ward S.R., Sundberg C.J., Chapman M.A. (2022). MRI characterization of skeletal muscle size and fatty infiltration in long-term trained and untrained individuals. Physiol. Rep..

[80] Nachit M., De Rudder M., Thissen J.P., Schakman O., Bouzin C., Horsmans Y., Vande Velde G., Leclercq I.A. (2021). Myosteatosis rather than sarcopenia associates with non-alcoholic steatohepatitis in non-alcoholic fatty liver disease preclinical models. J. Cachexia Sarcopenia Muscle.

[81] Pasmans K., Adriaens M.E., Olinga P., Langen R., Rensen S.S., Schaap F.G., Olde Damink S.W.M., Caiment F., van Loon L.J.C., Blaak E.E. (2021). Hepatic steatosis contributes to the development of muscle atrophy via inter-organ crosstalk. Front. Endocrinol..

[82] Guo S., Feng Y., Zhu X., Zhang X., Wang H., Wang R., Zhang Q., Li Y., Ren Y., Gao X. (2023). Metabolic crosstalk between skeletal muscle cells and liver through IRF4-FSTL1 in nonalcoholic steatohepatitis. Nat. Commun..

[83] Samuel V.T., Petersen K.F., Shulman G.I. (2010). Lipid-induced insulin resistance: Unravelling the mechanism. Lancet.

[84] Petersen K.F., Dufour S., Befroy D., Garcia R., Shulman G.I. (2004). Impaired mitochondrial activity in the insulin-resistant offspring of patients with type 2 diabetes. N. Engl. J. Med..

[85] Petersen K.F., Dufour S., Savage D.B., Bilz S., Solomon G., Yonemitsu S., Cline G.W., Befroy D., Zemany L., Kahn B.B. (2007). The role of skeletal muscle insulin resistance in the pathogenesis of the metabolic syndrome. Proc. Natl. Acad. Sci. USA.

[86] Goodpaster B.H., Carlson C.L., Visser M., Kelley D.E., Scherzinger A., Harris T.B., Stamm E., Newman A.B. (2001). Attenuation of skeletal muscle and strength in the elderly: The Health ABC Study. J. Appl. Physiol..

[87] Chien S.C., Chiu H.C., Chiu Y.C., Wang R.-H., Dillera K.P.O., Lee K.-T., Tsai H.-W., Tsai Y.-S., Ou H.-Y., Cheng P.-N. (2025). Clinical relevancies of sarcopenic obesity in patients with metabolic dysfunction-associated fatty liver disease (MASLD). Dig. Dis. Sci..

[88] Channapragada T., Stine J.G. (2025). Sarcopenic obesity and MASLD: A dangerous duo driving deadly cardiometabolic risk. Dig. Dis. Sci..

[89] Chen M., Cao Y., Ji G., Zhang L. (2023). Lean nonalcoholic fatty liver disease and sarcopenia. Front. Endocrinol..

[90] Hui J.M., Hodge A., Farrell G.C., Kench J.G., Kriketos A., George J. (2004). Beyond insulin resistance in NASH: TNF-α or adiponectin?. Hepatology.

[91] Tandon P., Montaño-Loza A.J., Lai J.C., Dasarathy S., Merli M. (2021). Sarcopenia and frailty in decompensated cirrhosis. J. Hepatol..

[92] Bhanji R.A., Narayanan P., Moynagh M.R., Takahashi N., Angirekula M., Kennedy C.C., Mara K.C., Dierkhising R.A., Watt K.D. (2019). Differing impact of sarcopenia and frailty in nonalcoholic steatohepatitis and alcoholic liver disease. Liver Transpl..

[93] Jiang M.J., Wu M.C., Duan Z.H., Wu J., Xu X.T., Li J., Meng Q.H. (2024). Prevalence and clinical impact of sarcopenia in liver transplant recipients: A meta-analysis. World J. Gastroenterol..

[94] Palmer B.F., Clegg D.J. (2015). The sexual dimorphism of obesity. Mol. Cell. Endocrinol..

[95] Harrison S.A., Bedossa P., Guy C.D., Schattenberg J.M., Loomba R., Taub R., Labriola D., Moussa S.E., Neff G.W., Rinella M.E. (2024). A phase 3, randomized, controlled trial of resmetirom in NASH with liver fibrosis. N. Engl. J. Med..

[96] U.S. Food and Drug Administration (2024). FDA Approves First Treatment for Patients with Liver Scarring due to Fatty Liver Disease. https://www.fda.gov/news-events/press-announcements/fda-approves-first-treatment-patients-liver-scarring-due-fatty-liver-disease.

[97] Sanyal A.J., Newsome P.N., Kliers I., Østergaard L.H., Long M.T., Kjær M.S., Cali A.M.G., Bugianesi E., Rinella M.E., Roden M. (2025). Phase 3 trial of semaglutide in metabolic dysfunction-associated steatohepatitis. N. Engl. J. Med..

[98] U.S. Food and Drug Administration (2025). FDA Approves Treatment for Serious Liver Disease Known as MASH. https://www.fda.gov/drugs/news-events-human-drugs/fda-approves-treatment-serious-liver-disease-known-mash.

[99] Loomba R., Hartman M.L., Lawitz E.J., Vuppalanchi R., Boursier J., Bugianesi E., Yoneda M., Behling C., Cummings O.W., Tang Y. (2024). Tirzepatide for metabolic dysfunction-associated steatohepatitis with liver fibrosis. N. Engl. J. Med..

[100] Souza M., Al-Sharif L., Antunes V.L.J., Huang D.Q., Loomba R. (2025). Comparison of pharmacological therapies in metabolic dysfunction-associated steatohepatitis for fibrosis regression and MASH resolution: Systematic review and network meta-analysis. Hepatology.

[101] Tripathi R., Gupta S., Jarnes L., Zeidan N., Tao M., Jamorabo D. Efficacy of GLP-1 receptor agonists in MASH resolution and fibrosis reduction: A meta-analysis. Abstract P3746. Proceedings of the ACG 2025 Annual Scientific Meeting.

[102] Lassailly G., Caiazzo R., Ntandja-Wandji L.-C., Gnemmi V., Baud G., Verkindt H., Ningarhari M., Louvet A., Leteurtre E., Raverdy V. (2020). Bariatric surgery provides long-term resolution of nonalcoholic steatohepatitis and regression of fibrosis. Gastroenterology.

[103] Cusi K., Orsak B., Bril F., Lomonaco R., Hecht J., Ortiz-Lopez C., Tio F., Hardies J., Darland C., Musi N. (2016). Long-term pioglitazone treatment for patients with nonalcoholic steatohepatitis and prediabetes or type 2 diabetes mellitus. Ann. Intern. Med..

[104] Loomba R., Sanyal A.J., Kowdley K.V., Bhatt D.L., Alkhouri N., Frias J.P., Bedossa P., Harrison S.A., Lazas D., Barish R. (2023). Randomized, controlled trial of the FGF21 analogue pegozafermin in NASH. N. Engl. J. Med..

[105] Ferrannini E., Baldi S., Frascerra S., Astiarraga B., Heise T., Bizzotto R., Mari A., Pieber T.R., Muscelli E. (2016). Shift to fatty substrate utilization in response to sodium-glucose cotransporter 2 inhibition in subjects without diabetes and patients with type 2 diabetes. Diabetes.

[106] Belfort R., Harrison S.A., Brown K., Darland C., Finch J., Hardies J., Balas B., Gastaldelli A., Tio F., Pulcini J. (2006). A placebo-controlled trial of pioglitazone in subjects with non-alcoholic steatohepatitis. N. Engl. J. Med..

[107] Tran Q.T., Bui T.T., Ngo L.T., Yang B.R., Baek I.H., Nguyen V.H., Lee K.A., Yun H.Y., Chae J.W., Lee S. (2025). Model-Based Meta-Analysis of the Relationship Between Pioglitazone and Histological Outcomes in Metabolic Dysfunction-Associated Steatohepatitis Patients. CPT Pharmacomet. Syst. Pharmacol..

[108] Tontonoz P., Spiegelman B.M. (2008). Fat and beyond: The diverse biology of PPARγ. Annu. Rev. Biochem..

[109] Lehmann J.M., Moore L.B., Smith-Oliver T.A., Wilkison W.O., Willson T.M., Kliewer S.A. (1995). An antidiabetic thiazolidinedione is a high affinity ligand for peroxisome proliferator-activated receptor γ (PPARγ). J. Biol. Chem..

[110] Kharitonenkov A., Shiyanova T.L., Koester A., Ford A.M., Micanovic R., Galbreath E.J., Sandusky G.E., Hammond L.J., Moyers J.S., Owens R.A. (2005). FGF-21 as a novel metabolic regulator. J. Clin. Investig..

[111] Badman M.K., Pissios P., Kennedy A.R., Koukos G., Flier J.S., Maratos-Flier E. (2007). Hepatic fibroblast growth factor 21 is regulated by PPARalpha and is a key mediator of hepatic lipid metabolism in ketotic states. Cell Metab..

[112] Harrison S.A., Dubourg J. (2024). FGF21 agonists: An emerging therapeutic for metabolic dysfunction-associated steatohepatitis and beyond. J. Hepatol..

[113] BonDurant L.D., Ameka M., Naber M.C., Markan K.R., Idiga S.O., Acevedo M.R., Walsh S.A., Ornitz D.M., Potthoff M.J. (2017). FGF21 regulates metabolism through adipose-dependent and -independent mechanisms. Cell Metab..

[114] Lin Z., Tian H., Lam K.S.L., Lin S., Hoo R.C., Konishi M., Itoh N., Wang Y., Bornstein S.R., Xu A. (2013). Adiponectin mediates the metabolic effects of FGF21 on glucose homeostasis and insulin sensitivity in mice. Cell Metab..

[115] Fisher F.M., Kleiner S., Douris N., Fox E.C., Mepani R.J., Verdeguer F., Wu J., Kharitonenkov A., Flier J.S., Maratos-Flier E. (2012). FGF21 regulates PGC-1α and browning of white adipose tissues in adaptive thermogenesis. Genes Dev..

[116] Holland W.L., Adams A.C., Brozinick J.T., Bui H.H., Miyauchi Y., Kusminski C.M., Bauer S.M., Wade M., Singhal E., Cheng C.C. (2013). An FGF21-adiponectin-ceramide axis controls energy expenditure and insulin action in mice. Cell Metab..

[117] Harrison S.A., Frias J.P., Neff G., Abrams G.A., Lucas K.J., Sanchez W., Gogia S., Sheikh M.Y., Behling C., Bedossa P. (2023). Safety and efficacy of once-weekly efruxifermin versus placebo in non-alcoholic steatohepatitis (HARMONY): A multicentre, randomised, double-blind, placebo-controlled, phase 2b trial. Lancet Gastroenterol. Hepatol..

[118] Noureddin M., Frias J.P., Neff G.W., Lucas K.J., Behling C., Bedossa P., Dubourg J., Chan D., Burch M., Fong E. (2025). Safety and efficacy of once-weekly efruxifermin versus placebo in metabolic dysfunction-associated steatohepatitis (HARMONY): 96-week results from a multicentre, randomised, double-blind, placebo-controlled, phase 2b trial. Lancet.

[119] Newsome P.N., Buchholtz K., Cusi K., Linder M., Okanoue T., Ratziu V., Sanyal A.J., Sejling A.-S., Harrison S.A. (2021). A placebo-controlled trial of subcutaneous semaglutide in nonalcoholic steatohepatitis. N. Engl. J. Med..

[120] Caussy C., Cusi K., Rosenstock J., Bugianesi E., Thomas M.K., Tang Y., Mather K.J., Loomba R., Sanyal A.J., Hartman M.L. (2025). Relationship between metabolic and histological responses in people with metabolic dysfunction-associated steatohepatitis with and without type 2 diabetes: Participant-level exploratory analysis of the SYNERGY-NASH trial with tirzepatide. Diabetes Care.

[121] Drucker D.J. (2018). Mechanisms of action and therapeutic application of glucagon-like peptide-1. Cell Metab..

[122] Vendrell J., El Bekay R., Peral B., García-Fuentes E., Megia A., Macias-Gonzalez M., Fernández Real J., Jimenez-Gomez Y., Escoté X., Pachón G. (2011). Study of the potential association of adipose tissue GLP-1 receptor with obesity and insulin resistance. Endocrinology.

[123] Pyke C., Heller R.S., Kirk R.K., Ørskov C., Reedtz-Runge S., Kaastrup P., Hvelplund A., Bardram L., Calatayud D., Knudsen L.B. (2014). GLP-1 receptor localization in monkey and human tissue: Novel distribution revealed with extensively validated monoclonal antibody. Endocrinology.

[124] Nishimiya N., Tajima K., Imajo K., Kameda A., Yoshida E., Togashi Y., Aoki K., Inoue T., Nakajima A., Utsunomiya D. (2021). Effects of canagliflozin on hepatic steatosis, visceral fat and skeletal muscle among patients with type 2 diabetes and non-alcoholic fatty liver disease. Intern. Med..

[125] Tokubuchi I., Tajiri Y., Iwata S., Hara K., Wada N., Hashinaga T., Nakayama H., Mifune H., Yamada K. (2017). Beneficial effects of metformin on energy metabolism and visceral fat volume through a possible mechanism of fatty acid oxidation in human subjects and rats. PLoS ONE.

[126] Huang X., Luo M., Zeng Y., Yi J., Lin S., Wang Y., Zheng X., Luo X. (2025). Effect of therapeutic lifestyle changes on patients with overweight/obesity and non-alcoholic fatty liver disease: A randomized controlled trial. Am. J. Med. Sci..

[127] Noureddin M., Rinella M., Taub R., Labriola D., Camacho R.C., Alkhouri N., Loomba R., Bansal M.B. (2025). Effects of resmetirom on metabolic dysfunction-associated steatohepatitis in patients with weight loss and/or diabetes taking glucagon-like peptide-1 receptor agonists and other diabetes therapies: A secondary analysis of the MAESTRO-NASH trial. Aliment. Pharmacol. Ther..

[128] Sinha R.A., Singh B.K., Yen P.M. (2018). Direct effects of thyroid hormones on hepatic lipid metabolism. Nat. Rev. Endocrinol..

[129] Kannt A., Wohlfart P., Madsen A.N., Veidal S.S., Feigh M., Schmoll D. (2021). Activation of thyroid hormone receptor-β improved disease activity and metabolism independent of body weight in a mouse model of non-alcoholic steatohepatitis and fibrosis. Br. J. Pharmacol..

[130] Kelly M.J., Pietranico-Cole S., Larigan J.D., Haynes N.E., Reynolds C.H., Scott N., Vermeulen J., Dvorozniak M., Conde-Knape K., Huang K.S. (2014). Discovery of 2-[3,5-dichloro-4-(5-isopropyl-6-oxo-1,6-dihydropyridazin-3-yloxy)phenyl]-3,5-dioxo-2,3,4,5-tetrahydro [1,2,4]triazine-6-carbonitrile (MGL-3196), a highly selective thyroid hormone receptor β agonist in clinical trials for the treatment of dyslipidemia. J. Med. Chem..

[131] Mátis D., Hegyi P., Teutsch B., Tornai T., Erőss B., Pár G., Váncsa S. (2023). Improved body composition decreases the fat content in non-alcoholic fatty liver disease: A meta-analysis and systematic review of longitudinal studies. Front. Med..

[132] Lee S., Kim K.W., Lee J., Park T., Park H.J., Song G.-W., Lee S.-G. (2021). Reduction of visceral adiposity as a predictor for resolution of nonalcoholic fatty liver in potential living liver donors. Liver Transpl..

